# Supplementary material for: Randomised controlled trial of a person-centred transition programme for adolescents with type 1 diabetes (STEPSTONES-DIAB): a study protocol
Source: BMJ Open. 2020 Apr 14;10(4):e036496. doi: 10.1136/bmjopen-2019-036496 (PMC7200039; doi:10.1136/bmjopen-2019-036496)
Supplement: Supplementary data [file bmjopen-2019-036496supp001.pdf]

| Variable | Measurements | Items | Validity | Reliability | Responsiveness | Interpretation |
|---|---|---|---|---|---|---|
| **Primary Outcome** | | | | | | |
| *Patient empowerment* [29] | Gothenburg Young Persons Empowerment Scale (GYPES) | 15 | Preliminary evidence for content validity and structural validity in young persons with CHD and diabetes. Results from CFA demonstrated that in addition to the subscale scores, an overall empowerment score can be validly computed. | Internal consistency confirmed in young persons with CHD and diabetes with α values of 0.858 for the overall scale and over 0.6 for all subscales.). | As a proxy for assessment of responsiveness, one of the participants had the lowest scoring, only 2.2% had the highest possible score. | Score from 15-75. Higher score reflecting a higher level of patient empowerment. |
| **Secondary Outcomes** | | | | | | |
| *Transition readiness* [38] | Readiness for Transition Questionnaire (RTQ) adolescent version | 26 | Validity based on relationships with other variables confirmed in young persons with kidney transplant[38] | Internal consistency confirmed in young persons with kidney transplant with α values over 0.70 [38] | Not reported | Scores from 10-40. Higher scores denote increased adolescent or caregiver responsibility. |
| *Transition readiness* [38] | Readiness for Transition Questionnaire (RTQ) parent version | 26 | Construct validity reported for parents of young persons with kidney transplant. | Reliability reported for parents of young persons with kidney transplant showed α values over 0.70. | Not reported | Scores from 10-40. Higher scores denote increased adolescent or caregiver responsibility. |
| *Health behaviors* [41] | Health Behavior Scale-Congenital Heart Disease (HBS-CHD) | 15 | Item content validity, scale content validity index and validity based on relationships to other variables confirmed in adolescents with CHD. Items deemed relevant for diabetes. | Stability not confirmed. | Confirmed in adolescents with CHD by Guyatt's Responsiveness Index. | Substance use, dental hygiene and total health risk score from 0-100. Physical exercise score from 0-100. |
| *Diabetes burden* [33] | Check your health | Perceived physical and emotional health, social relations and general QoL on four vertical scales | Face validity confirmed in adolescents with diabetes, good discriminant validity and moderate convergent validity | Test-retest reliability moderate to good, (i.e. correlation coefficient >0.7) Perceived QoL, emotional health and QoL without diabetes had correlation coefficients just below 0.7. | No floor effects on any of the four health dimensions (0%), ceiling effect (people scoring 100) ranged from 5% to 30%. | Four scales ranging from 0 to 100, higher scores indicating better perceived health ⁄QoL. |

| | | | | | | |
|---|---|---|---|---|---|---|
| *HRQOL* [35 36] | DisabKids Chronic Generic Measure-12 | 12 | Construct validity as well as convergent and discriminant validity of the DCGM-37 confirmed. [35]<br><br>No information found on validity for the short version. | Good reliability was found in a Swedish population of children with diabetes, α values of 0.932. Test-retest ICC coefficients were 0.913 and split-half correlation was 0.930. [36]<br><br>No information found on reliability for the short version. | None reached the floor of the scale. In total, 6% of the children answered at the top of a scale. [36] | Total score is 12–60. Higher scores indicating better HRQOL. |
| *Level of control* [36 37] | Disabkids Diabetes Module-10 | 10 | Face and content validity confirmed in children with diabetes.[37] | Internal consistency confirmed in young persons with diabetes with α values of 0.83 and 0.84 for the two subscales.[37] Confirmed in a Swedish population, α values of 0.85, test-retest ICC coefficients were 0.913 and split-half correlation 0.848. [36] | None reached the floor of the scale. In total, 6% of the children answered at the top of a scale.[36] | Total score is 10–50. Higher scores indicate higher level of control. |
| *Parental uncertainty* [39] | Linear analog scale (LAS) | 1<br>+ alternative option: "I have not thought about the transfer" | Face validity confirmed in parents to adolescents with CHD. | Under investigation | | Score is from 0 (very uncertain about the transfer to adult care) to 100 (not uncertain about the transfer of care). |
| *Satisfaction with care* | Transitional Care Experiences Questionnaire | 17<br>+ 3 open ended question | Under investigation | | | Score from 17-85. Higher score reflecting a higher level of satisfaction with transitional care |



Brorsson AL, *et al. BMJ Open* 2020; 10:e036496. doi: 10.1136/bmjopen-2019-036496
