# Supplementary material for: Randomised controlled trial of a person-centred transition programme for adolescents with type 1 diabetes (STEPSTONES-DIAB): a study protocol
Source: BMJ Open. 2020 Apr 14;10(4):e036496. doi: 10.1136/bmjopen-2019-036496 (PMC7200039; doi:10.1136/bmjopen-2019-036496)
Supplement: Author's manuscript [file bmjopen-2019-036496.draft_revisions.pdf]

BMJ Open is committed to open peer review. As part of this commitment we make the peer review history of every article we publish publicly available.

When an article is published we post the peer reviewers' comments and the authors' responses online. We also post the versions of the paper that were used during peer review. These are the versions that the peer review comments apply to.

The versions of the paper that follow are the versions that were submitted during the peer review process. They are not the versions of record or the final published versions. They should not be cited or distributed as the published version of this manuscript.

BMJ Open is an open access journal and the full, final, typeset and author-corrected version of record of the manuscript is available on our site with no access controls, subscription charges or pay-per-view fees (http://bmjopen.bmj.com).

If you have any questions on BMJ Open's open peer review process please email info.bmjopen@bmj.com

BMJ Open

A randomized controlled trial of a person-centred transition program for adolescents with type 1 diabetes (STEPSTONES-DIAB) – a study protocol

Journal:	*BMJ Open*
Manuscript ID	bmjopen-2019-036496
Article Type:	Protocol
Date Submitted by the Author:	17-Dec-2019
Complete List of Authors:	Brorsson, Anna Lena; Karolinska Institutet, Department of Neurobiology, Care Sciences and Society; University of Gothenburg, Gothenburg Centre for Person-Centred Care (GPCC)<br>Bratt, Ewa-Lena; University of Gothenburg, Institute of Health and Care Sciences; The Queen Silvia Children's Hospital, Department of Pediatric Cardiology<br>Moons, Philip; KU Leuven, Department of Public Health and Primary Care; University of Gothenburg, Institute of Health ans Care Sciences<br>Ek, Anna ; Karolinska Institutet, Division of Pediatrics, Department of Clinical Science, Intervention and Technology; Karolinska University Hospital, Department of Paediatric Diabetes, Astrid Lindgren's Children's Hospital<br>Jelleryd, Elisabeth ; Karolinska University Hospital, Department of Paediatric Diabetes, Astrid Lindgren's Children's Hospital<br>Torbjörnsdotter, Torun; Karolinska University Hospital, Department of Paediatric Diabetes, Astrid Lindgren's Children's Hospital<br>Sparud-Lundin, Carina; University of Gothenburg, Institute of Health and Care Sciences; University of Gothenburg, Gothenburg Centre for Person-Centred Care (GPCC)
Keywords:	DIABETES & ENDOCRINOLOGY, MEDICAL EDUCATION & TRAINING, PAEDIATRICS

SCHOLARONE™
Manuscripts

I, the Submitting Author has the right to grant and does grant on behalf of all authors of the Work (as defined in the below author licence), an exclusive licence and/or a non-exclusive licence for contributions from authors who are: i) UK Crown employees; ii) where BMJ has agreed a CC-BY licence shall apply, and/or iii) in accordance with the terms applicable for US Federal Government officers or employees acting as part of their official duties; on a worldwide, perpetual, irrevocable, royalty-free basis to BMJ Publishing Group Ltd ("BMJ") its licensees and where the relevant Journal is co-owned by BMJ to the co-owners of the Journal, to publish the Work in this journal and any other BMJ products and to exploit all rights, as set out in our licence.

The Submitting Author accepts and understands that any supply made under these terms is made by BMJ to the Submitting Author unless you are acting as an employee on behalf of your employer or a postgraduate student of an affiliated institution which is paying any applicable article publishing charge ("APC") for Open Access articles. Where the Submitting Author wishes to make the Work available on an Open Access basis (and intends to pay the relevant APC), the terms of reuse of such Open Access shall be governed by a Creative Commons licence – details of these licences and which Creative Commons licence will apply to this Work are set out in our licence referred to above.

Other than as permitted in any relevant BMJ Author's Self Archiving Policies, I confirm this Work has not been accepted for publication elsewhere, is not being considered for publication elsewhere and does not duplicate material already published. I confirm all authors consent to publication of this Work and authorise the granting of this licence.

For peer review only

A randomized controlled trial of a person-centred transition program for adolescents with type 1 diabetes (STEPSTONES-DIAB) – a study protocol

Anna Lena Brorsson[1,2], Ewa-Lena Bratt[3,4], Philip Moons[3,5,6], Anna Ek[7,8], Elisabeth Jelleryd[8], Torun Torbjörnsdotter[8], Carina Sparud-Lundin[2,3]

1. Department of Neurobiology, Care Sciences and Society, Karolinska Institutet, Stockholm, Sweden
2. Gothenburg Centre for Person-Centred Care (GPCC), University of Gothenburg, Gothenburg, Sweden
3. Institute of Health and Care Sciences, University of Gothenburg, Gothenburg, Sweden
4. Department of Pediatric Cardiology, The Queen Silvia Children's Hospital, Gothenburg, Sweden
5. Department of Public Health and Primary Care, KU Leuven, Leuven, Belgium
6. Department of Paediatrics and Child Health, University of Cape Town, South Africa
7. Division of Pediatrics, Department of Clinical Science, Intervention and Technology, Karolinska Institutet, Stockholm, Sweden
8. Department of Paediatric Diabetes, Astrid Lindgren's Children's Hospital, Karolinska University Hospital, Stockholm, Sweden

Corresponding author: Anna Lena Brorsson, Department of Neurobiology, Care Sciences and Society, Karolinska Institutet, Alfred Nobels allé 23, C3, SE-141 83 Huddinge, Sweden, Tel +46 70 961 5987, mail anna-lena.brorsson@ki.se.

For peer review only

For peer review only - http://bmjopen.bmj.com/site/about/guidelines.xhtml

Email addresses:

ELB: ewa-lena.bratt@gu.se

PM: philip.moons@kuleuven.be

AE: anna.ek.1@ki.se

EJ: elisabeth.jelleryd@sll.se

TT: torun.torbjornsdotter@sll.se

CSL: carina.s-lundin@fhs.gu.se

Word count: 5296

Keywords: Adolescent; Person centred care; Randomized controlled trial; Health Transition, Diabetes Mellitus, Type 1

For peer review only

ABSTRACT

Introduction: Adolescence is a critical period for youths with chronic conditions, when they are supposed to take over the responsibility for their health. Type 1 diabetes (T1D) is one of the most common chronic conditions in childhood and inadequate self-management increases the risk of short- and long-term complications. There is a lack of evidence regarding the effectiveness of transition programs. As a part of the STEPSTONES research program, the objective of this study is to evaluate the effectiveness and experiences of different transitional care models, including a person-centred transition program aiming to empower adolescents with T1D to become active partners in their health and care.

Methods and analysis: In this randomized controlled trial, patients are recruited from two paediatric diabetes clinics at the age of 16 years. Patients are randomly assigned to either the intervention group (n=70) where they will receive usual care plus the structured transition program, or to the control group (n=70) where they will only receive usual care. Data will be collected at 16, 17 and 18.5 years of age. In a later stage, the intervention group will be compared with adolescents in a dedicated youth clinic in a third setting. The primary outcome is patient empowerment. Secondary outcomes include generic, diabetes-specific and transfer-specific variables.

Ethics and dissemination: The study has been approved by the Ethical Review Board in Stockholm (Dnr 2018/1725-31). Findings will be reported following the CONSORT statement and disseminated in peer-reviewed journals and at international conferences.

Trial registration number: NCT03994536 (RCT)

For peer review only

ARTICLE SUMMARY

Strengths and limitations of this study

- The evidence of transition interventions targeting different long-term conditions is still insufficient; this study will expand current knowledge on transitional care
- Experience from an ongoing project where the person-centred transition program is evaluated in another group of adolescents with long-term conditions assures study fidelity
- Blinding of participants is not possible
- Long-term follow-up of the effectiveness of transition interventions are important and desirable. Although not included in this study, prolonged follow-up is needed.

For peer review only

INTRODUCTION

Worldwide, nearly 132 600 000 children and adolescents under 20 years develop type 1 diabetes (T1D) each year. The annual mean incidence rate varies from 0.1-60/100 000, with Sweden as one of the "top" incidence countries.[1] Children with T1D need life-long medical follow-up to prevent acute and long-term complications. The life-long follow up will comprise a shift in care when adolescents are transferred from a pediatric to adult care services. Simultaneously there is a shift in roles between parents and adolescents, where the adolescent needs to start taking over the responsibility for their health and care. Furthermore, this shift occurs during a vulnerable developmental period, when adolescents are exposed to unique challenges.[2 3] A smooth transition to adulthood and well-timed and planned transfer to adult care allows adolescents to optimize their ability to assume adult roles and functioning, which can improve medical outcome.[4] In this article, transfer is defined as the actual event when adolescents move from a pediatric to adult care services, while transition is defined as the process by which they become prepared to take charge of their lives and their health in adulthood.[5]

Transition programs for adolescents with chronic conditions aim to prepare and support them during the transition to adulthood and transfer to adult care so that they can gradually become active partners in their health and care. In this respect, adequate self-management and participation in care are essential and it is of utmost importance that patients feel empowered.[6] Patient empowerment is a critical attribute of person-centred care (PCC) and highlights the importance of knowing the person behind the patient.[7] Patient empowerment is considered to be *"An enabling process or outcome arising from communication with the healthcare professional and a mutual sharing of resources over information relating to illness, which enhances the patient's feelings of control, self-efficacy, coping abilities and ability to achieve change over their condition"*.[8] Research on the application of patient empowerment in

For peer review only - http://bmjopen.bmj.com/site/about/guidelines.xhtml

adolescent health and transitional care is limited. The application of PCC principles in young persons with chronic diseases is unprecedented, although there is a growing demand for PCC in diabetes care. Moreover, research on transition and transfer and on interventions that have the potential to improve both processes is important. Nevertheless, evidence on the effectiveness of transition programs using a randomized controlled design (RCT) is sparingly reported.[9] Evidence base for interventions that improve outcomes for this specific target group is limited and not adequate in guiding clinical practice to support better self-management and outcomes in youths with T1D.[10]

It is known from previous studies that transfer from pediatric to adult care services is associated with deterioration in health of adolescents with certain chronic conditions.[9] For adolescents with T1D this includes non-optimal glycemic control and increased diabetes related hospitalizations after transfer, as well as general patient dissatisfaction with the transition experience. Poor clinic attendance after transfer is considered a risk factor.[11 12] Potential importance of continuity of care, support, education and individualized support is highlighted across different types of interventions targeting young persons with T1D.[13] Within diabetes care, health care professionals agree that a lack of an effective and structured transfer from pediatric to adult care may contribute to insufficient continuity of health care and increased risk of adverse outcomes in young adults with T1D.[14 15]

To investigate the effectiveness of a structured person-centred transition program for adolescents with chronic conditions, the STEPSTONES (Swedish Transition Effects Project Supporting Teenagers with chrONic mEdical conditionS) has been established. STEPSTONES is a research project in which a transition program has been developed and currently tested in an ongoing RCT in adolescents with congenital heart

For peer review only - http://bmjopen.bmj.com/site/about/guidelines.xhtml

disease (CHD).[16 17] The STEPSTONES transition program is generic in nature and can therefore be expanded to other childhood-onset conditions, such as T1D.

Objectives and hypothesis

The overall objective of STEPSTONES-DIAB is to evaluate the effectiveness of transitional care, aiming to empower adolescents with T1D to become active partners in their health and care.

The following hypotheses will be tested in two steps:

1. Adolescents with T1D who receive a structured, person-centred transition program have a higher patient empowerment score than adolescents who receive usual care.
2. A structured, person-centred transition program is equivalent to a dedicated youth clinic in empowering adolescents with T1D.

METHODS AND ANALYSIS

We have used the recommendations in the SPIRIT reporting guidelines in this protocol paper.[18]

Design and setting

An RCT is being conducted in which patients are randomly assigned to either the intervention group (study arm 1) where they receive usual care plus the structured transition program, or to the control group (study arm 2) where they receive usual care only. This study is being conducted in two paediatric diabetes clinics. In parallel, all patients followed-up in a special youth clinic comprise study arm 3, to be compared with study arm 1 at a later stage. All the clinics are located at University Hospitals in Stockholm, Sweden. The number of transferred patients per year differs between the centres. For an overview of the three arms, see Figure 1.

For peer review only - http://bmjopen.bmj.com/site/about/guidelines.xhtml
For peer review only

Participants and recruitment

Inclusion criteria for the study:

- Literate, Swedish-speaking adolescents with T1D
- Aged 16 years
- Diabetes duration > 1 year
- If other diagnoses are present, T1D must be the primary diagnosis and not a side effect of other diagnoses or treatment e.g. cystic fibrosis or cortisone-triggered diabetes.

Parents/guardians, as well as the adolescents will be asked to participate, the inclusion criterion being that they are literate and Swedish-speaking. Participants will be excluded if they are diagnosed with conditions affecting cognitive abilities and may have difficulties understanding and/or completing questionnaires.

A transition coordinator (TC) will contact 16-year-old adolescents eligible for inclusion in the RCT during scheduled outpatient visits or by telephone. The adolescents and their guardians will be informed both verbally and in writing about the purpose of the study, its voluntary nature, the right to cease participation at any time, the data storage procedure, and the strict anonymous processing of data. In the youth clinic (study arm 3), a data collection officer (DCO) will handle recruitment and data collection.

At both RCT centres, participants are assigned to either the intervention or the control group. This is done with a web-based randomization system (1:1) employing stratified block randomization with a random variable block size. Block randomization ensures that the TCs in the two centres have relatively continuous exposure to the intervention over time, allowing them to keep their skills up to date and to ensure the fidelity of the intervention. The fact that the block sizes vary randomly minimizes the possibility of the TCs being able to

For peer review only

predict group assignments. Stratification is made by the centre to achieve an equal distribution of patients in the intervention, with control groups at both centres. Furthermore, stratification for sex and HbA1c is crucial to ensure the distribution in the groups due to the knowledge that female adolescents have poorer glycemic control compared with males, and that adolescents with unsatisfactory glycemic control are a high-risk population.[19 20] The grouping for HbA1c is based on ISPAD guidelines 2014.[19] Overall, this approach decreases the within-centre variability and reduces the risk of bias and confounding.[21] An intention-to-treat principle will be used.

Intervention

Transition programme (intervention group)

A multi-component intervention, adapted from the brief transition program of Hilderson et al.[22] and the STEPSTONES-CHD project [16] will be implemented. This transition program comprises eight key components: (1) a transition coordinator (TC); (2) a person-centred written transfer plan; (3) provision of information and education about T1D and treatment, health behaviours, dealing with school, friends, leisure activities and the importance of treatment adherence; (4) high access to the TC via telephone/sms; (5) information about and contact with the adult diabetes care team; (6) meeting with other young people with T1D; (7) support for and guidance of parents; and (8) the actual transfer to the adult diabetes outpatient clinic. The components are implemented in five steps: (1) first visit with TC at the diabetes pediatric outpatient clinic at the age of 16 years; (2) second visit with TC at the diabetes pediatric outpatient clinic at the age of 17 years; (3) "adolescent day" group information with parents and adolescents, with the participation of healthcare professionals from the adult outpatient clinic and young adults with T1D; (4) third visit with TC at the diabetes outpatient clinic at the age of 18 years; and (5) the current transfer to one of the eight adult outpatient

For peer review only - http://bmjopen.bmj.com/site/about/guidelines.xhtml

clinics that care for adults with T1D in the area (Figure 1). The intervention is delivered by specialized trained pediatric nurses at the pediatric diabetes outpatient clinic. The content of the patient education is adapted to T1D.

Usual care (intervention and control group)

The current usual care is provided to all participants, irrespective of whether they are randomized to the intervention or not. Usual care includes check-up visits to the paediatric diabetes outpatient clinic according to international, national and local guidelines every third months, with adjustments for individual needs.[23 24] In both centres, visits include a meeting at the pediatric diabetes outpatient clinic with a nurse or a physician. It is up to each family if the parents attend these visits. In one of the intervention centres, no targeted transitional care interventions will be implemented. In the other two centres, usual care also includes that some adolescents (planned to be transferred to the adult diabetes outpatient clinic in the same hospital) will be scheduled for a transfer visit in groups. They will be asked to write an individual referral letter including information on: occupation, interests, alcohol and tobacco habits, latest HbA1c, estimation of how everyday life with diabetes works (estimated on a linear analogue scale (0-10), goals with diabetes treatment, what they want help with and what they remember from when they were diagnosed. Thereafter, the group of adolescents will visit the adult outpatient diabetes clinic (accompanied by a pediatric nurse or a pediatrician) to receive information from the adult care professionals and an allocated time for their first individual appointment. If an adolescent does not want to participate in this group referral, the same routines apply as for the rest of the adolescents at the pediatric clinic, i.e., they will be referred directly to the chosen adult diabetes clinic. None of the included centres have a formal TC in place and no additional transfer preparation.

For peer review only - http://bmjopen.bmj.com/site/about/guidelines.xhtml

Youth clinic

At the dedicated youth clinic (study arm 3), adolescents with T1D will be followed up from 13 years of age and a more flexible transfer to adult care at 19-20 years of age will be applied. Usual care in this clinic includes visits to the diabetes nurse or the physician at the unit every second to third month with closer visits if needed, according to the patient's glycemic control. Midwives and social workers are employed at the unit and, if needed, visits to these can be arranged. No formal TC is in place, nor a formalized transition/transfer plan.

For peer review only

Intervention fidelity

The special training of TC together with their experience of working with children and adolescents with T1D will ensure compliance that the components are implemented in line with the developed intervention. Prior to the start of this study, the TCs received five days of training including: adolescent psychosocial development and health with focus on chronic diseases in general and T1D in particular; PCC and empowerment; common neuropsychiatric diagnoses; the legal and human rights of young people. Moreover, they received training in communicating and interviewing young people using the psychosocial interview guide HEEADS, the acronym standing for home, education/employment, activities, drugs, sexuality and suicide/depression. The method uses structured questions to facilitate communication and create a sympathetic, confidential, respectful environment.[25] The TC training was provided by experts within each field. TCs were also introduced to the administrative parts of the study in line with Good Clinical Practice (GCP) and have participated in a course in GCP.

Standard Operating Procedures (SOP) were developed to ensure consistency throughout the study. These SOPs include ethical approval, study preparation and study administration, participant recruitment and survey completion; usual care, data storage, entry and security, study progress, process evaluation and publication policy. To avoid

For peer review only - http://bmjopen.bmj.com/site/about/guidelines.xhtml

contamination, the TCs will not care for adolescents from the control group or adolescents eligible for the study, and team members at the outpatient clinic who are not involved in the study will only be given a brief outline of the intervention program content.

Data collection

Baseline data will be collected when participants (the adolescent and one or two parents) have agreed to participate in the study. Participants are asked to complete the set of pen-and-paper questionnaires at 16, 17 and 18.5 years of age (Figure 1). These are sent by post to the participants' home address before the next scheduled outpatient visit. The pack contains an instruction letter, a set of questionnaires and a prepaid and addressed reply envelope. Baseline data (T0) will be collected before any intervention is started (study arm 1). For T0, the most recent HbA1c value before inclusion in the study will be used. It is estimated that the questionnaires will take approximately 30 minutes to complete. Participants will be asked to complete the set of questionnaires themselves and to return them within three weeks. If the questionnaires are not returned within this time frame, patients will be reminded by a new set of questionnaires or asked to complete them while waiting in the waiting room at the next visit. [16 26]

Outcome measures

The questionnaire obtained at T0 provides background information including: sex, age, education level, born in Sweden or not; adolescent/parents born in Sweden or not; living situation (adolescent)/marital status (guardian); number and order of siblings. For guardians, employment is also required.

For peer review only - http://bmjopen.bmj.com/site/about/guidelines.xhtml

Primary outcome measures

Empowerment

The primary outcome is empowerment. Empowerment is measured with the Gothenburg Young Persons Empowerment Scale (GYPES).[27] This scale has been tested in a cross-sectional study in order to determine its psychometric properties in adolescents with CHD and T1D and supporting GYPES validity and reliability as a tool for assessing young persons' empowerment.[26] The five subscales measure: (a) self-perceived level of understanding of their disease (knowledge and understanding); (b) the capacity patients have to handle their disease (personal control); (c) the effect their illness has on their lives and sense of self (identity); (d) the capacity to make decisions along with the healthcare professional (shared decision-making); and (e) the ability to share their experiences and help others who are going through a similar situation (enabling others). The primary outcome, analysed at T2, is the total score ranging from 15 to 75 points, with a higher score reflecting a higher level of empowerment.[27 28]

Secondary outcome measures

The secondary outcome variables chosen are other important developmental tasks in adolescence and emerging adulthood and in relation to T1D. When evaluating interventions aimed at improving the outcome of young adults with T1D, some recommended core outcomes are set.[29] These eight outcomes are: measures of diabetes-related burden or stress, diabetes-related quality of life, number of severe hypoglycemic events, self-management behaviour; number of instances of diabetic ketoacidosis (DKA), objectively-measured glycated hemoglobin (HbA1C), level of clinic engagement and perceived level of control over diabetes. In addition, we measure indicators for successful transition and transfer outcomes as proposed by Coyne et al.,[30] which include clinic attendance, hospitalization rates, disease-specific outcomes and transfer-specific satisfaction. See

For peer review only - http://bmjopen.bmj.com/site/about/guidelines.xhtml

Table 1 for an overview of included variables and measurements. Secondary outcomes include endpoint at T2 and change from baseline to T2, while the subscales in questionnaires will be included in exploratory analyses.

Generic

Health behaviours are measured with the Health Behaviour Scale. This questionnaire has been validated in adolescents with CHD but is also considered to be valid for the diabetes group. Health behaviours are 'activities a person undertakes to maintain or improve health and prevent diseases. This scale assesses alcohol consumption, tobacco use, dental care and physical activity. The scale has 15 items that help to calculate three summary risk scores: substance use (score 0–100), dental hygiene (score 0–100) and total health risk score (score 0–100). Higher risk scores represent unhealthier behaviors.[31]

Diabetes specific

Diabetes duration, Insulin administration (Multiple Daily Injections, MDI or Continuous Subcutaneous Insulin Infusion, CSII), Blood glucose testing (Self-Monitoring Blood Glucose testing, SMBG/Continuous Glucose Monitoring CGM/Intermittent scanning Continuous Glucose Monitoring, isCGM) are descriptive variables. Hypoglycemic events and diabetic ketoacidosis (DKA) will be summed up for the whole study period and compared at T2. HbA1c will be analysed using DCA Vantage (Siemens Healthcare Diagnostics AB, Upplands Väsby, Sweden) and the test results collected from the National Diabetes Register (NDR). Analyses will be performed at T2 and change from baseline to T2.

'Check your Health' measures self-perceived physical and emotional health, social relationships and general QoL on four vertical thermometer scales ranging from 0 to

For peer review only

100, with 0 indicating low self-perceived health. Each scale indicates self-perceived health with diabetes and, on the same scale, self-perceived health imagined without diabetes. The measured difference between self-perceived health with and the imagined without diabetes is defined as the burden of diabetes. [32]

DISABKIDS Chronic Generic Measure - DCGM-12 measures general quality of life and the level of distress caused by a chronic disease. The short-form of the DISABKIDS chronic generic module consists of 12 five-point Likert-scaled items assigned to the three domains: mental, social and physical. Higher scores represent better outcome.[33-35]

DISABKIDS Diabetes Module (DM) consists of 13 five-point Likert scaled items and has two scales: an Impact and a Treatment scale. The Impact scale describes emotional reactions to needing to control everyday life and to restricting one's diet. The Treatment scale refers to carrying equipment and planning treatment. Higher scores represent better outcome.[33 34 36]

Transfer specific

The Readiness for transition questionnaire RTQ (adolescent and parent version) examines two aspects. Firstly, the overall transition readiness is assessed, using two items ranging from 1 to 4. The sum of these two items results in a score ranging from 2 to 8. Secondly, the frequency of adolescent responsibility and parental involvement are reported for 10 different health behaviours on a four-point Likert scale. Each variable results in a total score ranging from 10 to 40, with higher scores indicating higher adolescent responsibility or parental involvement respectively.[37]

Parental uncertainty is measured using a 10 cm Linear Analogue Scale (LAS) with the end points 'not uncertain at all' (=0) and 'extremely uncertain' (=100). Parental

For peer review only - http://bmjopen.bmj.com/site/about/guidelines.xhtml

uncertainty comprehends parents' perceptions of certainty/uncertainty prior to their child's transfer to adult care. This LAS has been used in a cross-sectional study of adolescents with CHD, was developed for an ongoing study in the STEPSTONES-CHD project and has been used in a cross-sectional study.[38]

The new questionnaire on transfer and transition experiences is under construction and will be tested on young persons aged 19-21 years with long-term conditions and experience of the transfer to adult care in order to evaluate its psychometric properties.

Clinical indicators, such as attendance for scheduled outpatient visits before and after transfer to adult clinic and the duration between last visit in pediatric care and the first in adult care will be collected from the medical record.

All instruments have been demonstrated to have good validity, reliability and responsiveness.[27 31-38]

Table 1 Overview of variables and measurements in Stepstones-DIAB

**Variables/indicators**	**Measurement**	**Time point**	**Source**
**PRIMARY OUTCOME**			
Patient empowerment	Gothenburg Young Persons Empowerment scale (GYPES-DIAB) [27]	T0, T1, T2	A
**SECONDARY OUTCOMES**			
**Generic**			
Health behavior	Health Behavior Scale [39]	T0, T1, T2	A
**Diabetes-specific**	Diabetes duration<br>Insulin administration (MDI/CSII)<br>Blood glucose testing (SMBG/CGM/isCGM)	T0	NDR
Glycemic control	HbA1c, hypoglycemic events, diabetic ketoacidosis	T0, T1, T2	NDR
Diabetes burden	Check your health [32]	T0, T1, T2	A
HRQoL /level of control	DisabKids Chronic Generic Measure-12 [33-35]<br>Disabkids Diabetes Module-10 [33 34 36]	T0, T1, T2	A
**Transfer-specific**			

For peer review only - http://bmjopen.bmj.com/site/about/guidelines.xhtml

Transition readiness	Readiness for Transition Questionnaire (RTQ) [37]	T0, T1, T2	A + P
Uncertainty (parents)	Uncertainty Scale (Linear Analog Scale) [38]	T0, T1, T2	P
Clinical indicators	Clinic attendance rates, pre- and post-transfer<br>Time between last visit in PC and first visit in AC	T2	Admin
Transitional Care experiences Questionnaire	In progress	T2	A

A= Adolescents, P= Parents, NDR= National Diabetes Registry, PC=Pediatric care, AC= Adult care

Data management

The completed questionnaires are entered into a secure research database, using a web-based data entry system, accessible for research group only. Each patient is assigned a unique study code and this code will be used as a unique identifier in the database to ensure patient anonymity. The list of study codes and the list of eligible participants will be stored separately in a secure file to which only the TC coordinators have access. In study arm 3, a data collection officer will handle this process.

Data monitoring and quality checks will be undertaken by a team member with vast experience of database management. To make data collection procedures uniform, TCs and the DCO have been provided with thorough training; regular visits will be made by the project coordinator and regular quality assurance sessions will be organized for TCs and DCO.

Owing to the nature of the intervention and the study design, it is not possible to blind the participants but TCs in charge of carrying out the intervention and data collection are not involved in the preparation of the intervention design or in the statistical analyses.

For peer review only

Proposed sample size

Based on the primary outcome, we target an improved patient empowerment score of 5.25 points on a scale from 15-75 (i.e. 0.5 SD).[26] For two-sided tests with alpha=0.05 and power=80%, 63 patients are needed in each arm of the RCT. In order to compensate for a potential 10% dropout rate, 70 patients will be included in each arm. Given that the two pediatric diabetes outpatient clinics together have about 100 patients in each age cohort of adolescents, and considering a 70% participation rate, a 24-month recruitment period is needed to enrol 140 patients in study arm 1 and 2.

Data analysis

The primary endpoint (total score of the level of empowerment at T2) will be analysed using Fisher's non-parametric permutation test unadjusted between the intervention group and the control group in the RCT on the Full Analysis Set with imputation of missing values using stochastic imputation. A sensitivity analysis will be conducted with ANCOVA adjusting for stratification variables without imputation. If confounders are found, complementary analyses will be conducted using ANCOVA between the two groups adjusted for these confounders. A confounder is a baseline variable that differs between the groups and influences the primary outcome variable. For comparison between the two randomized groups, Fisher's non-parametric permutation test will be used for continuous variables, Fisher's exact test for dichotomous variables, Mantel-Haenszel chi-square test for ordered categorical variables and Pearson Chi-square test for non-ordered categorical variables. Missing data will be handled with stochastic imputation. Mean difference between the two groups with 95% CI will be presented for all continuous and dichotomous variables. For comparison within groups, Fisher's non-parametric permutation test for paired observation will be used for continuous variables and Sign test for dichotomous variables and ordered categorical variables.

For peer review only - http://bmjopen.bmj.com/site/about/guidelines.xhtml

All analyses will be predefined in a detailed statistical analysis plan (SAP) before data base lock. In a later stage, a comparison between the intervention group and a youth clinic group (study arm 3) will also be performed. All significance tests will be two-sided and conducted at the 5% significance level.

Patient and public involvement

During the planning phase of this study, young persons from the Swedish patient organization, “Young Diabetes”, have been involved as advisors in the development of certain parts of the intervention. Insufficient transitional care has been on their agenda for several years and the initiative to evaluate support for transition and transfer is therefore highly sought after. Representatives from Young Diabetes will continuously be involved as a reference group during the entire study to secure consideration of young peoples’ priorities, experiences and preferences in all phases. These representatives also participate in the event “Adolescent Day”. There is an established advisory board in STEPSTONES that has been involved in the development of the intervention, also including young persons with CHD and parent representatives.[27] Henceforth, this advisory board will serve the entire STEPSTONES project, and thus young persons with diabetes have been included in the advisory board. Further, this group includes experts on adolescent health and medicine, as well as on the research and implementation of PCC.

Trial duration

Recruitment of participants began in August 2019 and is expected to be completed in August 2021. Data collection is planned to be completed in January 2024. When data collection is completed, the dataset will be closed and the analysis of data and the dissemination of the results will begin.

For peer review only - http://bmjopen.bmj.com/site/about/guidelines.xhtml

Ethical considerations

For this study, Ethics approval has been obtained from the Regional Ethics Review Board in Stockholm (Dnr 2018/1725-31). A multi-centre ethical application includes mandatory approvals from the head of the department at each participating centre.

Before inclusion, an information pack is given to the adolescents and their parents to explain the purpose of the study, its voluntary nature, the right to cease participation at any time, the data storage procedure, and the strict anonymous processing of data. Patients will only be included if the adolescent, who is a minor at the time of inclusion, and the parents/guardians provide written informed consent.

Personal data regarding patients' health will be collected, and patients given the chance to be exposed to a new intervention that is different from current usual care. The risk of participating in this study is low, as the intervention is supportive in nature. If the transition programme is shown to be effective, the benefits may be substantial. We consider the participants to be vulnerable persons. For some participants, filling out the questionnaires may bring up some unpleasant memories and/or feelings, so contact details of the TCs transition coordinators/data collection officers, as well as organisations which can help them cope with these feelings, will be provided in the information pack. Participants are covered by Swedish patient insurance in case of adverse events.

Follow-up on adverse events

All adverse events (AE) will be reported within a week to the principal investigator and the project manager. In case of a serious AE, the report will be made immediately. Appropriate measures will be undertaken in every AE to ensure patent safety.

For peer review only

Dissemination

The results from this study will be published in peer-reviewed journals and will follow the recommendations of the CONSORT statement.[40] Further, abstracts for poster and oral presentation will be submitted to national and international conferences and findings communicated and discussed with health care and patient representatives for potential implementation.

DISCUSSION

This study relies on the experiences from two previous research projects aiming to promote the transition process for youths. The first instance was for adolescents with rheumatoid arthritis, the second is the current STEPSTONES-CHD research project by testing a further developed transition program in adolescents with CHD ,[16 41] and now we are expanding to evaluate the effectiveness of a structured, person-centred transition programme for adolescents with T1D. The focus is to increase their level of empowerment in order to promote their ability to navigate through the adult care system, participate in care planning, and increase self-management and decision-making skills.[6 7]

As previously stated, creating effective clinical processes for the transition from pediatric to adult care is particularly important in order to optimize well-being and health in emerging adults with T1D. This includes achieving target glycemic control to prevent long-term complications and to maximize lifelong functioning. Although there is a lack of proven strategies to achieve these goals, programs that particularly target adolescents with T1D through education, skills training, special transition clinics, and/or addition of TCs appear to be promising.[10 42] However, the current knowledge regarding this transition process calls for

For peer review only

ongoing and expanding research initiatives in order to determine the effectiveness of transition programs for adolescents with different long term conditions.[9 43]

One of the strengths of this study is the chosen design; employing an RCT is considered the gold standard when evaluating the effectiveness of interventions and provides the highest level of evidence. By using stratified block randomisation, we ensure that the intervention and control groups are balanced in terms of potential confounding factors, e.g. equal distribution, thereby minimising the risk of biasing the result. In a previous empowerment-based study including a person-centred group education for youths with T1D, the results highlighted the importance of an approach that strengthens young people's independence and autonomy. The parents' attitude was also highlighted in this process. The adolescents described the importance of a permissive atmosphere and that it was perceived as positive to meet at new healthcare professional in these encounters. Moreover, the adolescents said it was empowering to meet others with T1D.[44] For HbA1c, an effect could only be seen for boys.[45] Another strength is that projects can use the lessons learned from STEPSTONES-CHD in terms of feasibility and fidelity. For example, experiences of standard operating procedures for recruitment and data collection will secure data entry. Moreover, in the training of the TCs, the experienced TCs from STEPSTONES-CHD could be used as tutors and mentors, which further secures delivery of the generic intervention components in STEPSTONES-DIAB. By employing experienced diabetes nurses as TCs, we ensure that the generic components are adapted to and relevant for diabetes care. Further, the generic components in the person-centred transition program, as well as the generic outcome measures, provide opportunities to compare findings across different conditions. If the program is proven effective, these circumstances may facilitate future implementation of transfer and transition strategies in clinical practice.

For peer review only - http://bmjopen.bmj.com/site/about/guidelines.xhtml

A challenge in complex interventions like transition programs is the multiple interacting components and often challenging behaviours required by those delivering and receiving the intervention. To evaluate the full extent of a complex intervention in addition to effectiveness, the evaluation must incorporate process assessments of how the intervention is being delivered in practice and what causal mechanisms lead to the desired outcomes. Based on the experiences from the more extensive process evaluation undertaken in STEPSTONES-CHD,[17] we have identified certain process evaluation components that also need to be undertaken in STEPSTONES-DIAB in order to promote future implementation. These components reflect what is delivered (fidelity, adherence, dose, reach, acceptability) and are provided through participant observations, the intervention implementation form, enrolment form and transition plans.[17]

Some limitations need to be acknowledged in this study. Firstly, this intervention focuses on transition to adulthood, the actual transfer to adult care and, to a lesser extent, changes in the adult care services. Additional interventions may be needed to secure continuity of care after transfer. Secondly, the lack of blinding procedures poses a limitation in that the nature of study makes blinding patients and TCs impossible. Thirdly, the short follow-up period of six months at the age of 18.5 years cannot be considered enough to evaluate long-term effects of the transition program. There is a great need to evaluate such effects[9] and these must be followed up in separate studies after additional consent and funding.

CONCLUSION

The empirical underpinning of the guidelines, recommendations and statements regarding successful transitional strategies is weak and to be considered expert-based rather than evidence-based. Based on an RCT design and building on previous projects, STEPSTONES-

For peer review only - http://bmjopen.bmj.com/site/about/guidelines.xhtml

DIAB has the research capacity to provide high-level evidence within this area, and thereby contribute to filling the gaps in scientific knowledge related to transitional care of adolescents with T1D.

Author contributions

CSL, ELB, PM and ALB designed the study. ALB and CSL drafted the manuscript. All authors read and approved the final manuscript.

Funding statement

This work was supported by the Swedish Child Diabetes Foundation (no grant number available), the Swedish Diabetes Association Research Foundation (grant DIA2018-326), Swedish Research Council for Health, Working Life and Welfare-FORTE (grant STYA-2015/0003), Swedish Research Council (grant 2015-02503), the Institute of Health and Care Sciences of the University of Gothenburg and the Gothenburg University Centre for Person-centred care (GPCC).

Competing interests

None declared

For peer review only

REFERENCES

1. International Diabetes Federation (IDF). IDF Diabetes Atlas, 8th edition. 2017
2. Michaud PA, Suris JC, Viner R. The adolescent with a chronic condition. Part II: healthcare provision. *Arch Dis Child* 2004;89(10):943-9. doi: 10.1136/adc.2003.045377 [published Online First: 2004/09/24]
3. Colver A, Longwell S. New understanding of adolescent brain development: relevance to transitional healthcare for young people with long term conditions. *Arch Dis Child* 2013;98(11):902-7. doi: 10.1136/archdischild-2013-303945 [published Online First: 2013/08/30]
4. American Academy of Pediatrics, American Academy of Family Physicians, American College of Physicians-American Society of Internal Medicine. A consensus statement on health care transitions for young adults with special health care needs. *Pediatrics* 2002;110(6 Pt 2):1304-6. [published Online First: 2002/11/29]
5. Suris JC, Belanger RE, Ambresin AE, et al. Extra burden of psychosomatic complaints among adolescents suffering from chronic conditions. *J Dev Behav Pediatr* 2011;32(4):328-31. doi: 10.1097/DBP.0b013e3181fa5727 [published Online First: 2011/04/20]
6. Bravo P, Edwards A, Barr PJ, et al. Conceptualising patient empowerment: a mixed methods study. *BMC Health Serv Res* 2015;15:252. doi: 10.1186/s12913-015-0907-z
7. Ekman I, Swedberg K, Taft C, et al. Person-centered care--ready for prime time. *Eur J Cardiovasc Nurs* 2011;10(4):248-51. doi: S1474-5151(11)00129-0 [pii] 10.1016/j.ejcnurse.2011.06.008 [published Online First: 2011/07/19]
8. Small N, Bower P, Chew-Graham CA, et al. Patient empowerment in long-term conditions: development and preliminary testing of a new measure. *BMC Health Serv Res* 2013;13:263. doi: 10.1186/1472-6963-13-263 [published Online First: 2013/07/10]
9. Campbell F, Biggs K, Aldiss SK, et al. Transition of care for adolescents from paediatric services to adult health services. *The Cochrane database of systematic reviews* 2016;4:Cd009794. doi: 10.1002/14651858.CD009794.pub2 [published Online First: 2016/04/30]
10. Schultz AT, Smaldone A. Components of Interventions That Improve Transitions to Adult Care for Adolescents With Type 1 Diabetes. *J Adolesc Health* 2017;60(2):133-46. doi: 10.1016/j.jadohealth.2016.10.002 [published Online First: 2016/12/13]
11. Castensoe-Seidenfaden P, Jensen AK, Smedegaard H, et al. Clinical, behavioural and social indicators for poor glycaemic control around the time of transfer to adult care: a longitudinal study of 126 young people with diabetes. *Diabet Med* 2017;34(5):667-75. doi: 10.1111/dme.13318 [published Online First: 2017/01/19]
12. Shulman R, Chafe R, Guttmann A. Transition to Adult Diabetes Care: A Description of Practice in the Ontario Pediatric Diabetes Network. *Canadian journal of diabetes* 2019;43(4):283-89. doi: 10.1016/j.jcjd.2018.10.007 [published Online First: 2019/02/06]
13. O'Hara MC, Hynes L, O'Donnell M, et al. A systematic review of interventions to improve outcomes for young adults with Type 1 diabetes. *Diabet Med* 2017;34(6):753-69. doi: 10.1111/dme.13276
14. Peters A, Laffel L. Diabetes care for emerging adults: recommendations for transition from pediatric to adult diabetes care systems: a position statement of the American Diabetes Association, with representation by the American College of Osteopathic Family Physicians, the American Academy of Pediatrics, the American Association of Clinical Endocrinologists, the American Osteopathic Association, the Centers for Disease Control and Prevention, Children with Diabetes, The Endocrine Society, the International Society for Pediatric and Adolescent Diabetes, Juvenile Diabetes Research Foundation International, the National Diabetes Education Program, and the Pediatric Endocrine Society (formerly Lawson Wilkins Pediatric Endocrine Society). *Diabetes Care* 2011;34(11):2477-85. doi: 10.2337/dc11-1723 [published Online First: 2011/10/26]

For peer review only

15. Cameron FJ, Garvey K, Hood KK, et al. ISPAD Clinical Practice Consensus Guidelines 2018: Diabetes in adolescence. *Pediatr Diabetes* 2018;19 Suppl 27:250-61. doi: 10.1111/pedi.12702 [published Online First: 2018/06/15]
16. Acuna Mora M, Sparud-Lundin C, Bratt EL, et al. Person-centred transition programme to empower adolescents with congenital heart disease in the transition to adulthood: a study protocol for a hybrid randomised controlled trial (STEPSTONES project). *BMJ open* 2017;7(4):e014593. doi: 10.1136/bmjopen-2016-014593 [published Online First: 2017/04/20]
17. Saarijarvi M, Wallin L, Moons P, et al. Transition program for adolescents with congenital heart disease in transition to adulthood: protocol for a mixed-method process evaluation study (the STEPSTONES project). *BMJ open* 2019;9(8):e028229. doi: 10.1136/bmjopen-2018-028229 [published Online First: 2019/08/05]
18. Chan AW, Tetzlaff JM, Altman DG, et al. SPIRIT 2013 Statement: defining standard protocol items for clinical trials. *Rev Panam Salud Publica* 2015;38(6):506-14. [published Online First: 2016/07/22]
19. Rewers MJ, Pillay K, de Beaufort C, et al. Assessment and monitoring of glycemic control in children and adolescents with diabetes. *Pediatr Diabetes* 2014;15 Suppl 20:102-14. doi: 10.1111/pedi.12190
20. The Swedish Pediatric Diabetes Quality Registry, SWEDIABKIDS. Swediabkids - The National Quality Register for Child and Adolescent Diabetes Annual Report 2018. 2019 [Available from: https://www.ndr.nu accessed 11 October 2019.
21. Efird J. Blocked randomization with randomly selected block sizes. *Int J Environ Res Public Health* 2011;8(1):15-20. doi: 10.3390/ijerph8010015 [published Online First: 2011/02/15]
22. Hilderson D, Westhovens R, Wouters C, et al. Rationale, design and baseline data of a mixed methods study examining the clinical impact of a brief transition programme for young people with juvenile idiopathic arthritis: the DON'T RETARD project. *BMJ open* 2013;3(12):e003591. doi: 10.1136/bmjopen-2013-003591 [published Online First: 2013/12/05]
23. Forsberg M, Forsander G, , Reference group. Diabetes at outpatient clinics for children and adolescents; Guidelines from the paediatrician association for endocrinology and diabetes in Sweden 2017 [Available from: http://endodiab.barnlakarforeningen.se/vardprogram/diabetes/ accessed 11 October 2019 2019.
24. Pihoker C, Forsander G, Fantahun B, et al. ISPAD Clinical Practice Consensus Guidelines 2018: The delivery of ambulatory diabetes care to children and adolescents with diabetes. *Pediatr Diabetes* 2018;19 Suppl 27:84-104. doi: 10.1111/pedi.12757 [published Online First: 2018/08/26]
25. Goldenring JM, E C. Getting into adolescent heads. *Contemp Pediatr* 1988
26. Apers S, Moons P, Goossens E, et al. Sense of coherence and perceived physical health explain the better quality of life in adolescents with congenital heart disease. *Eur J Cardiovasc Nurs* 2013;12(5):475-83. doi: 10.1177/1474515113477955 [published Online First: 2013/02/15]
27. Acuna Mora M, Luyckx K, Sparud-Lundin C, et al. Patient empowerment in young persons with chronic conditions: Psychometric properties of the Gothenburg Young Persons Empowerment Scale (GYPES). *PLoS One* 2018;13(7):e0201007. doi: 10.1371/journal.pone.0201007 [published Online First: 2018/07/22]
28. Acuna Mora M, Sparud-Lundin C, Burstrom A, et al. Patient empowerment and its correlates in young persons with congenital heart disease. *Eur J Cardiovasc Nurs* 2019;18(5):389-98. doi: 10.1177/1474515119835434 [published Online First: 2019/03/06]
29. Byrne M, O'Connell A, Egan AM, et al. A core outcomes set for clinical trials of interventions for young adults with type 1 diabetes: an international, multi-perspective Delphi consensus study. *Trials* 2017;18(1):602. doi: 10.1186/s13063-017-2364-y [published Online First: 2017/12/21]

For peer review only - http://bmjopen.bmj.com/site/about/guidelines.xhtml

30. Coyne B, Hallowell SC, Thompson M. Measurable Outcomes After Transfer From Pediatric to Adult Providers in Youth With Chronic Illness. *J Adolesc Health* 2017;60(1):3-16. doi: 10.1016/j.jadohealth.2016.07.006 [published Online First: 2016/09/12]
31. Goossens E, Luyckx K, Mommen N, et al. Health risk behaviors in adolescents and emerging adults with congenital heart disease: psychometric properties of the Health Behavior Scale-Congenital Heart Disease. *Eur J Cardiovasc Nurs* 2013;12(6):544-57. doi: 10.1177/1474515113475934
32. Viklund G, Wikblad K. Self-perceived health and burden of diabetes in teenagers with type 1 diabetes: psychometric properties of the Swedish measure 'check your health'. *Acta Paediatr* 2010;99(3):422-6. doi: APA1615 [pii]10.1111/j.1651-2227.2009.01615.x [published Online First: 2009/12/05]
33. Bullinger M, Schmidt S, Petersen C. Assessing quality of life of children with chronic health conditions and disabilities: a European approach. *Int J Rehabil Res* 2002;25(3):197-206. [published Online First: 2002/09/28]
34. Chaplin JE, Hanas R, Lind A, et al. Assessment of childhood diabetes-related quality-of-life in West Sweden. *Acta Paediatr* 2009;98(2):361-6. doi: 10.1111/j.1651-2227.2008.01066.x
35. Simeoni MC, Schmidt S, Muehlan H, et al. Field testing of a European quality of life instrument for children and adolescents with chronic conditions: the 37-item DISABKIDS Chronic Generic Module. *Qual Life Res* 2007;16(5):881-93. doi: 10.1007/s11136-007-9188-2 [published Online First: 2007/04/04]
36. Baars RM, Atherton CI, Koopman HM, et al. The European DISABKIDS project: development of seven condition-specific modules to measure health related quality of life in children and adolescents. *Health and quality of life outcomes* 2005;3:70. doi: 1477-7525-3-70 [pii] 10.1186/1477-7525-3-70 [published Online First: 2005/11/15]
37. Gilleland J, Amaral S, Mee L, et al. Getting ready to leave: transition readiness in adolescent kidney transplant recipients. *J Pediatr Psychol* 2012;37(1):85-96. doi: 10.1093/jpepsy/jsr049 [published Online First: 2011/09/01]
38. Burstrom A, Acuna Mora M, Ojmyr-Joelsson M, et al. Parental uncertainty about transferring their adolescent with congenital heart disease to adult care. *J Adv Nurs* 2019;75(2):380-87. doi: 10.1111/jan.13852 [published Online First: 2018/09/14]
39. Goossens E, Luyckx K, Mommen N, et al. Health risk behaviors in adolescents and emerging adults with congenital heart disease: psychometric properties of the Health Behavior Scale-Congenital Heart Disease. *Eur J Cardiovasc Nurs* 2013;12(6):544-57. doi: 10.1177/1474515113475934 [published Online First: 2013/01/31]
40. Moher D, Hopewell S, Schulz KF, et al. CONSORT 2010 explanation and elaboration: updated guidelines for reporting parallel group randomised trials. *Int J Surg* 2012;10(1):28-55. doi: 10.1016/j.ijsu.2011.10.001 [published Online First: 2011/11/01]
41. Hilderson D, Moons P, Van der Elst K, et al. The clinical impact of a brief transition programme for young people with juvenile idiopathic arthritis: results of the DON'T RETARD project. *Rheumatology (Oxford)* 2016;55(1):133-42. doi: 10.1093/rheumatology/kev284 [published Online First: 2015/09/01]
42. Gabriel P, McManus M, Rogers K, et al. Outcome Evidence for Structured Pediatric to Adult Health Care Transition Interventions: A Systematic Review. *J Pediatr* 2017;188:263-69.e15. doi: 10.1016/j.jpeds.2017.05.066 [published Online First: 2017/07/03]
43. Acuna Mora M, Saarijarvi M, Moons P, et al. The Scope of Research on Transfer and Transition in Young Persons With Chronic Conditions. *J Adolesc Health* 2019 doi: 10.1016/j.jadohealth.2019.07.014 [published Online First: 2019/09/22]
44. Brorsson AL, Lindholm Olinder A, Viklund G, et al. Adolescents' perceptions of participation in group education using the Guided Self-Determination-Young method: a qualitative study. *BMJ Open Diabetes Res Care* 2017;5(1):e000432. doi: 10.1136/bmjdrc-2017-000432 [published Online First: 2017/12/12]

For peer review only

45. Brorsson AL, Leksell J, Andersson Franko M, et al. A person-centered education for adolescents with type 1 diabetes-A randomized controlled trial. *Pediatr Diabetes* 2019 doi: 10.1111/pedi.12888 [published Online First: 2019/07/04]

For peer review only

For peer review only - http://bmjopen.bmj.com/site/about/guidelines.xhtml

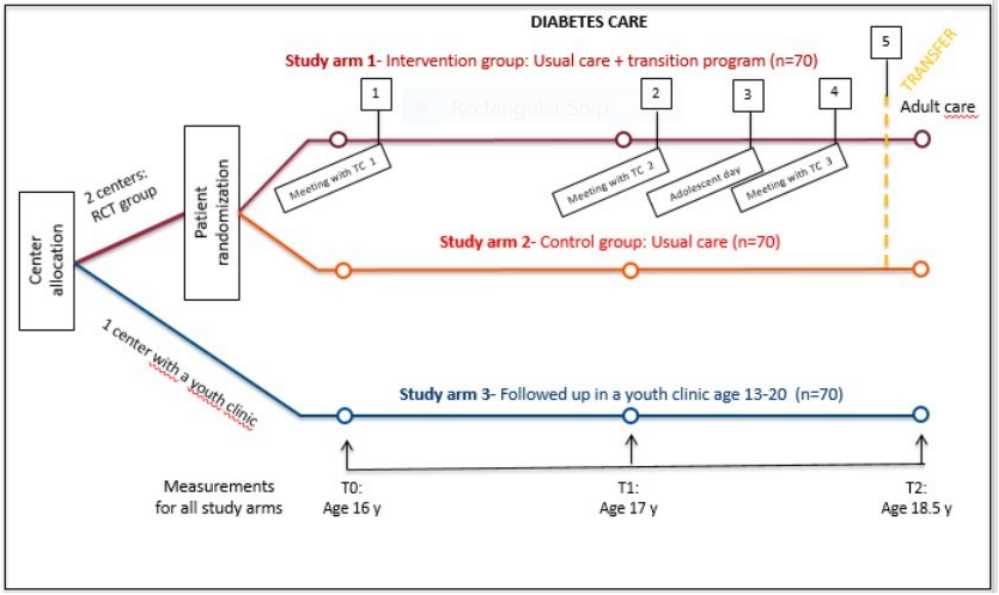

Figure 1 Overview of the study design

Reporting checklist for protocol of a clinical trial.

Based on the SPIRIT guidelines.

Instructions to authors

Complete this checklist by entering the page numbers from your manuscript where readers will find each of the items listed below.

Your article may not currently address all the items on the checklist. Please modify your text to include the missing information. If you are certain that an item does not apply, please write "n/a" and provide a short explanation.

Upload your completed checklist as an extra file when you submit to a journal.

In your methods section, say that you used the SPIRITreporting guidelines, and cite them as:

Chan A-W, Tetzlaff JM, Altman DG, Laupacis A, Gøtzsche PC, Krleža-Jerić K, Hróbjartsson A, Mann H, Dickersin K, Berlin J, Doré C, Parulekar W, Summerskill W, Groves T, Schulz K, Sox H, Rockhold FW, Rennie D, Moher D. SPIRIT 2013 Statement: Defining standard protocol items for clinical trials. Ann Intern Med. 2013;158(3):200-207

		Reporting Item	Page Number
**Administrative information**			
Title	#1	Descriptive title identifying the study design, population, interventions, and, if applicable, trial acronym	1
Trial registration	#2a	Trial identifier and registry name. If not yet registered, name of intended registry	3
Trial registration: data set	#2b	All items from the World Health Organization Trial Registration Data Set	7-21
Protocol version	#3	Date and version identifier	NA, First version
Funding	#4	Sources and types of financial, material, and other support	24

For peer review only

Roles and responsibilities: contributorship	#5a	Names, affiliations, and roles of protocol contributors	1-2, 24
Roles and responsibilities: sponsor contact information	#5b	Name and contact information for the trial sponsor	24, only funding agencies involved, no sponsors
Roles and responsibilities: sponsor and funder	#5c	Role of study sponsor and funders, if any, in study design; collection, management, analysis, and interpretation of data; writing of the report; and the decision to submit the report for publication, including whether they will have ultimate authority over any of these activities	24, funders have no role in these matters.
Roles and responsibilities: committees	#5d	Composition, roles, and responsibilities of the coordinating centre, steering committee, endpoint adjudication committee, data management team, and other individuals or groups overseeing the trial, if applicable (see Item 21a for data monitoring committee)	17, 19
**Introduction**			
Background and rationale	#6a	Description of research question and justification for undertaking the trial, including summary of relevant studies (published and unpublished) examining benefits and harms for each intervention	5-7
Background and rationale: choice of comparators	#6b	Explanation for choice of comparators	5-7
Objectives	#7	Specific objectives or hypotheses	7
Trial design	#8	Description of trial design including type of trial (eg, parallel group, crossover, factorial, single group), allocation ratio, and framework (eg, superiority, equivalence, non-inferiority, exploratory)	7-8

For peer review only

**Methods: Participants, interventions, and outcomes**			
Study setting	#9	Description of study settings (eg, community clinic, academic hospital) and list of countries where data will be collected. Reference to where list of study sites can be obtained	7
Eligibility criteria	#10	Inclusion and exclusion criteria for participants. If applicable, eligibility criteria for study centres and individuals who will perform the interventions (eg, surgeons, psychotherapists)	8, 10-12
Interventions: description	#11a	Interventions for each group with sufficient detail to allow replication, including how and when they will be administered	10-11
Interventions: modifications	#11b	Criteria for discontinuing or modifying allocated interventions for a given trial participant (eg, drug dose change in response to harms, participant request, or improving / worsening disease)	NA
Interventions: adherance	#11c	Strategies to improve adherence to intervention protocols, and any procedures for monitoring adherence (eg, drug tablet return; laboratory tests)	11
Interventions: concomitant care	#11d	Relevant concomitant care and interventions that are permitted or prohibited during the trial	7
Outcomes	#12	Primary, secondary, and other outcomes, including the specific measurement variable (eg, systolic blood pressure), analysis metric (eg, change from baseline, final value, time to event), method of aggregation (eg, median, proportion), and time point for each outcome. Explanation of the clinical relevance of chosen efficacy and harm outcomes is strongly recommended	12-17
Participant timeline	#13	Time schedule of enrolment, interventions (including any run-ins and washouts), assessments, and visits for participants. A	8-9 (Figure 1)

For peer review only

		schematic diagram is highly recommended (see Figure)	
Sample size	#14	Estimated number of participants needed to achieve study objectives and how it was determined, including clinical and statistical assumptions supporting any sample size calculations	18
Recruitment	#15	Strategies for achieving adequate participant enrolment to reach target sample size	8, 18
**Methods: Assignment of interventions (for controlled trials)**			
Allocation: sequence generation	#16a	Method of generating the allocation sequence (eg, computer-generated random numbers), and list of any factors for stratification. To reduce predictability of a random sequence, details of any planned restriction (eg, blocking) should be provided in a separate document that is unavailable to those who enrol participants or assign interventions	8-9
Allocation concealment mechanism	#16b	Mechanism of implementing the allocation sequence (eg, central telephone; sequentially numbered, opaque, sealed envelopes), describing any steps to conceal the sequence until interventions are assigned	8-9
Allocation: implementation	#16c	Who will generate the allocation sequence, who will enrol participants, and who will assign participants to interventions	8
Blinding (masking)	#17a	Who will be blinded after assignment to interventions (eg, trial participants, care providers, outcome assessors, data analysts), and how	18 (NA)
Blinding (masking): emergency unblinding	#17b	If blinded, circumstances under which unblinding is permissible, and procedure for revealing a participant’s allocated intervention during the trial	NA

For peer review only

**Methods: Data collection, management, and analysis**			
Data collection plan	#18a	Plans for assessment and collection of outcome, baseline, and other trial data, including any related processes to promote data quality (eg, duplicate measurements, training of assessors) and a description of study instruments (eg, questionnaires, laboratory tests) along with their reliability and validity, if known. Reference to where data collection forms can be found, if not in the protocol	12-17
Data collection plan: retention	#18b	Plans to promote participant retention and complete follow-up, including list of any outcome data to be collected for participants who discontinue or deviate from intervention protocols	12
Data management	#19	Plans for data entry, coding, security, and storage, including any related processes to promote data quality (eg, double data entry; range checks for data values). Reference to where details of data management procedures can be found, if not in the protocol	17
Statistics: outcomes	#20a	Statistical methods for analysing primary and secondary outcomes. Reference to where other details of the statistical analysis plan can be found, if not in the protocol	18-19
Statistics: additional analyses	#20b	Methods for any additional analyses (eg, subgroup and adjusted analyses)	18-19
Statistics: analysis population and missing data	#20c	Definition of analysis population relating to protocol non-adherence (eg, as randomised analysis), and any statistical methods to handle missing data (eg, multiple imputation)	18-19
**Methods: Monitoring**			

For peer review only

Data monitoring: formal committee	#21a	Composition of data monitoring committee (DMC); summary of its role and reporting structure; statement of whether it is independent from the sponsor and competing interests; and reference to where further details about its charter can be found, if not in the protocol. Alternatively, an explanation of why a DMC is not needed	NA
Data monitoring: interim analysis	#21b	Description of any interim analyses and stopping guidelines, including who will have access to these interim results and make the final decision to terminate the trial	NA
Harms	#22	Plans for collecting, assessing, reporting, and managing solicited and spontaneously reported adverse events and other unintended effects of trial interventions or trial conduct	20
Auditing	#23	Frequency and procedures for auditing trial conduct, if any, and whether the process will be independent from investigators and the sponsor	17, funders have no role in these matters.
**Ethics and dissemination**			
Research ethics approval	#24	Plans for seeking research ethics committee / institutional review board (REC / IRB) approval	20
Protocol amendments	#25	Plans for communicating important protocol modifications (eg, changes to eligibility criteria, outcomes, analyses) to relevant parties (eg, investigators, REC / IRBs, trial participants, trial registries, journals, regulators)	Protocol modifications will be communicated in the trial registry
Consent or assent	#26a	Who will obtain informed consent or assent from potential trial participants or authorised surrogates, and how (see Item 32)	8, 20
Consent or assent: ancillary studies	#26b	Additional consent provisions for collection and use of participant data and biological specimens in ancillary studies, if applicable	23

For peer review only

Confidentiality	#27	How personal information about potential and enrolled participants will be collected, shared, and maintained in order to protect confidentiality before, during, and after the trial	17
Declaration of interests	#28	Financial and other competing interests for principal investigators for the overall trial and each study site	24
Data access	#29	Statement of who will have access to the final trial dataset, and disclosure of contractual agreements that limit such access for investigators	Only members of the research group have access to the final dataset, as specified in the approved ethical application.
Ancillary and post trial care	#30	Provisions, if any, for ancillary and post-trial care, and for compensation to those who suffer harm from trial participation	NA
Dissemination policy: trial results	#31a	Plans for investigators and sponsor to communicate trial results to participants, healthcare professionals, the public, and other relevant groups (eg, via publication, reporting in results databases, or other data sharing arrangements), including any publication restrictions	21
Dissemination policy: authorship	#31b	Authorship eligibility guidelines and any intended use of professional writers	No
Dissemination policy: reproducible research	#31c	Plans, if any, for granting public access to the full protocol, participant-level dataset, and statistical code	No
**Appendices**			
Informed consent materials	#32	Model consent form and other related documentation given to participants and authorised surrogates	Only available in Swedish
Biological specimens	#33	Plans for collection, laboratory evaluation, and storage of biological specimens for genetic or	NA

For peer review only

molecular analysis in the current trial and for future use in ancillary studies, if applicable

None The SPIRIT checklist is distributed under the terms of the Creative Commons Attribution License CC-BY-ND 3.0. This checklist can be completed online using https://www.goodreports.org/, a tool made by the EQUATOR Network in collaboration with Penelope.ai

For peer review only

BMJ Open

A randomized controlled trial of a person-centred transition program for adolescents with type 1 diabetes (STEPSTONES-DIAB) – a study protocol

Journal:	*BMJ Open*
Manuscript ID	bmjopen-2019-036496.R1
Article Type:	Protocol
Date Submitted by the Author:	24-Feb-2020
Complete List of Authors:	Brorsson, Anna Lena; Karolinska Institutet, Department of Neurobiology, Care Sciences and Society; University of Gothenburg, Gothenburg Centre for Person-Centred Care (GPCC)<br>Bratt, Ewa-Lena; University of Gothenburg, Institute of Health and Care Sciences; The Queen Silvia Children's Hospital, Department of Pediatric Cardiology<br>Moons, Philip; KU Leuven, Department of Public Health and Primary Care; University of Gothenburg, Institute of Health ans Care Sciences<br>Ek, Anna ; Karolinska Institutet, Division of Pediatrics, Department of Clinical Science, Intervention and Technology; Karolinska University Hospital, Department of Paediatric Diabetes, Astrid Lindgren's Children's Hospital<br>Jelleryd, Elisabeth ; Karolinska University Hospital, Department of Paediatric Diabetes, Astrid Lindgren's Children's Hospital<br>Torbjörnsdotter, Torun; Karolinska University Hospital, Department of Paediatric Diabetes, Astrid Lindgren's Children's Hospital<br>Sparud-Lundin, Carina; University of Gothenburg, Institute of Health and Care Sciences; University of Gothenburg, Gothenburg Centre for Person-Centred Care (GPCC)
**Primary Subject Heading**:	Diabetes and endocrinology
Secondary Subject Heading:	Nursing, Paediatrics, Medical education and training
Keywords:	DIABETES & ENDOCRINOLOGY, MEDICAL EDUCATION & TRAINING, PAEDIATRICS

SCHOLARONE™
Manuscripts

I, the Submitting Author has the right to grant and does grant on behalf of all authors of the Work (as defined in the below author licence), an exclusive licence and/or a non-exclusive licence for contributions from authors who are: i) UK Crown employees; ii) where BMJ has agreed a CC-BY licence shall apply, and/or iii) in accordance with the terms applicable for US Federal Government officers or employees acting as part of their official duties; on a worldwide, perpetual, irrevocable, royalty-free basis to BMJ Publishing Group Ltd ("BMJ") its licensees and where the relevant Journal is co-owned by BMJ to the co-owners of the Journal, to publish the Work in this journal and any other BMJ products and to exploit all rights, as set out in our licence.

The Submitting Author accepts and understands that any supply made under these terms is made by BMJ to the Submitting Author unless you are acting as an employee on behalf of your employer or a postgraduate student of an affiliated institution which is paying any applicable article publishing charge ("APC") for Open Access articles. Where the Submitting Author wishes to make the Work available on an Open Access basis (and intends to pay the relevant APC), the terms of reuse of such Open Access shall be governed by a Creative Commons licence – details of these licences and which Creative Commons licence will apply to this Work are set out in our licence referred to above.

Other than as permitted in any relevant BMJ Author's Self Archiving Policies, I confirm this Work has not been accepted for publication elsewhere, is not being considered for publication elsewhere and does not duplicate material already published. I confirm all authors consent to publication of this Work and authorise the granting of this licence.

For peer review only

A randomized controlled trial of a person-centred transition program for adolescents with type 1 diabetes (STEPSTONES-DIAB) – a study protocol

Anna Lena Brorsson[1,2], Ewa-Lena Bratt[3,4], Philip Moons[2,3,5,6], Anna Ek[7,8], Elisabeth Jelleryd[8], Torun Torbjörnsdotter[8], Carina Sparud-Lundin[2,3]

1. Department of Neurobiology, Care Sciences and Society, Karolinska Institutet, Stockholm, Sweden
2. Gothenburg Centre for Person-Centred Care (GPCC), University of Gothenburg, Gothenburg, Sweden
3. Institute of Health and Care Sciences, University of Gothenburg, Gothenburg, Sweden
4. Department of Pediatric Cardiology, The Queen Silvia Children's Hospital, Gothenburg, Sweden
5. Department of Public Health and Primary Care, KU Leuven, Leuven, Belgium
6. Department of Paediatrics and Child Health, University of Cape Town, South Africa
7. Division of Pediatrics, Department of Clinical Science, Intervention and Technology, Karolinska Institutet, Stockholm, Sweden
8. Department of Paediatric Diabetes, Astrid Lindgren's Children's Hospital, Karolinska University Hospital, Stockholm, Sweden

Corresponding author: Anna Lena Brorsson, Department of Neurobiology, Care Sciences and Society, Karolinska Institutet, Alfred Nobels allé 23, C3, SE-141 83 Huddinge, Sweden, Tel +46 70 961 5987, mail anna-lena.brorsson@ki.se.

For peer review only

For peer review only - http://bmjopen.bmj.com/site/about/guidelines.xhtml

Email addresses:

ELB: ewa-lena.bratt@gu.se

PM: philip.moons@kuleuven.be

AE: anna.ek.1@ki.se

EJ: elisabeth.jelleryd@sll.se

TT: torun.torbjornsdotter@sll.se

CSL: carina.s-lundin@fhs.gu.se

Keywords:Adolescent; Diabetes Mellitus, Type 1; Health Transition; Person centred care; Randomized controlled trial;

For peer review only

ABSTRACT

Introduction: Adolescence is a critical period for youths with chronic conditions, when they are supposed to take over the responsibility for their health. Type 1 diabetes (T1D) is one of the most common chronic conditions in childhood and inadequate self-management increases the risk of short- and long-term complications. There is a lack of evidence regarding the effectiveness of transition programs. As a part of the STEPSTONES research program, the objective of this study is to evaluate the effectiveness and experiences of different transitional care models, including a person-centred transition program aiming to empower adolescents with T1D to become active partners in their health and care.

Methods and analysis: In this randomized controlled trial, patients are recruited from two paediatric diabetes clinics at the age of 16 years. Patients are randomly assigned to either the intervention group (n=70) where they will receive usual care plus the structured transition program, or to the control group (n=70) where they will only receive usual care. Data will be collected at 16, 17 and 18.5 years of age. In a later stage, the intervention group will be compared with adolescents in a dedicated youth clinic in a third setting. The primary outcome is patient empowerment. Secondary outcomes include generic, diabetes-specific and transfer-specific variables.

Ethics and dissemination: The study has been approved by the Ethical Review Board in Stockholm (Dnr 2018/1725-31). Findings will be reported following the CONSORT statement and disseminated in peer-reviewed journals and at international conferences.

Trial registration number:NCT03994536 (RCT)

For peer review only

ARTICLE SUMMARY

Strengths and limitations of this study

- The evidence of transition interventions targeting different long-term conditions is still insufficient; this study will expand current knowledge on transitional care
- Experience from an ongoing project where the person-centred transition program is evaluated in another group of adolescents with long-term conditions assures study fidelity
- Blinding of participants is not possible
- Long-term follow-up of the effectiveness of transition interventions are important and desirable. Although not included in this study, prolonged follow-up is needed.

For peer review only

INTRODUCTION

Worldwide, nearly 132 600 000 children and adolescents under 20 years develop type 1 diabetes (T1D) each year. The annual mean incidence rate varies from 0.1-60/100 000, with Sweden as one of the "top" incidence countries.[1]Children with T1D need life-long medical follow-up to prevent acute and long-term complications. The life-long follow up will comprise a shift in care when adolescents are transferred from a pediatric to adult care services. Simultaneously there is a shift in roles between parents and adolescents, where the adolescent needs to start taking over the responsibility for their health and care. Furthermore, this shift occurs during a vulnerable developmental period, when adolescents are exposed to unique challenges.[2 3]A smooth transition to adulthood and well-timed and planned transfer to adult care allows adolescents to optimize their ability to assume adult roles and functioning, which can improve medical outcome.[4]In this article, transfer is defined as the actual event when adolescents move from a pediatricto adult care services, while transition is defined as the process by which they become prepared to take charge of their lives and their health in adulthood.[5]

Transition programs for adolescents with chronic conditions aim to prepare and support them during the transition to adulthood and transfer to adult care so that they can gradually become active partners in their health and care. In this respect, adequate self-management and participation in care are essential and it is of utmost importance that patients feel empowered.[6]Patient empowerment is a critical attribute of person-centred care (PCC) and highlights the importance of knowing the person behind the patient.[7]Patient empowerment is considered to be *"An enabling process or outcome arising from communication with the healthcare professional and a mutual sharing of resources over information relating to illness, which enhances the patient's feelings of control, self-efficacy, coping abilities and ability to achieve change over their condition"*.[8]Research on the application of patient

For peer review only - http://bmjopen.bmj.com/site/about/guidelines.xhtml

empowerment in adolescent health and transitional care is limited. The application of PCC principles in young persons with chronic diseases is unprecedented, although there is a growing demand for PCC in diabetes care. Moreover, research on transition and transfer and on interventions that have the potential to improve both processes is important. Nevertheless, evidence on the effectiveness of transition programs using a randomized controlled design (RCT) is sparingly reported.[9]Evidence base for interventions that improve outcomes for this specific target group is limited and not adequate in guiding clinical practice to support better self-management and outcomes in youths with T1D.[10]

It is known from previous studies that transfer from pediatric to adult care services is associated with deterioration in health of adolescents with certain chronic conditions.[9]For adolescents with T1D this includes non-optimal glycemic control and increased diabetes related hospitalizations after transfer, as well as general patient dissatisfaction with the transition experience. Poor clinic attendance after transfer is considered a risk factor.[11 12]Potential importance of continuity of care, support, education and individualized support is highlighted across different types of interventions targeting young persons with T1D.[13]Within diabetes care, health care professionals agree that a lack of an effective and structured transfer from pediatric to adult care may contribute to insufficient continuity of health care and increased risk of adverse outcomes in young adults with T1D.[14 15]

To investigate the effectiveness of a structured person-centred transition program for adolescents with chronic conditions, the STEPSTONES (Swedish Transition Effects Project Supporting Teenagers with chrONicmEdicalconditionS) has been established. STEPSTONES is a research project in which a transition program has been developed and currently tested in an ongoing RCT in adolescents with congenital heart disease (CHD).[16 17] Theoretical framework for the transition program is supported by the conceptualization of

For peer review only - http://bmjopen.bmj.com/site/about/guidelines.xhtml

empowerment defined by Small et al [8] and with other attributes related to the concept. The components are following the philosophy of PCC, [7] operationalized, and guided by several change theories/models described by Acuna Mora et al.[18]The STEPSTONES transition program is generic in nature and can therefore be expanded to other childhood-onset conditions, such as T1D.

Objectives and hypothesis

The overall objective of STEPSTONES-DIAB is to evaluate the effectiveness of transitional care, aiming to empower adolescents with T1D to become active partners in their health and care.

The following hypotheses will be tested in two steps:

1. Adolescents with T1D who receive a structured, person-centred transition program have a higher patient empowerment score than adolescents who receive usual care.
2. A structured, person-centred transition program is equivalent to a dedicated youth clinic in empowering adolescents with T1D.

METHODS AND ANALYSIS

We have used the recommendations in the SPIRIT reporting guidelines in this protocol paper.[19]

Design and setting

An RCT is being conducted in two paediatric diabetes clinics in which patients are randomly assigned to either the intervention group (study arm 1) where they receive usual care plus the structured transition program, or to the control group (study arm 2) where they receive usual care only. In parallel, all patients followed-up in a special youth clinic comprise study arm 3,

For peer review only

to be compared with study arm 1 at a later stage. All the clinics are located at University Hospitals in Stockholm, Sweden. The number of transferred patients per year differs between the centres. For an overview of the three study arms, see Figure 1.

Participants and recruitment

Inclusion criteria for the study:

- Literate, Swedish-speaking adolescents with T1D
- Aged 16 years
- Diabetes duration > 1 year
- If other diagnoses are present, T1D must be the primary diagnosis and not a side effect of other diagnoses or treatment e.g. cystic fibrosis or cortisone-triggered diabetes.

Parents/guardians, as well as the adolescents will be asked to participate, the inclusion criterion being that they are literate and Swedish-speaking. Participants will be excluded if they are diagnosed with conditions affecting cognitive abilities and may have difficulties understanding and/or completing questionnaires.

A transition coordinator (TC) will contact 16-year-old adolescents eligible for inclusion in the RCT (study arm 1 and 2) during scheduled outpatient visits or by telephone. The adolescents and their guardians will be informed both verbally and in writing about the purpose of the study, its voluntary nature, the right to cease participation at any time, the data storage procedure, and the strict anonymous processing of data. In the youth clinic (study arm 3), a data collection officer (DCO) will handle recruitment and data collection.

At both RCT centres, participants are assigned to either the intervention or the control group. This is done with a web-based randomization system (1:1) employing stratified block randomization with a random variable block size. Block randomization ensures that the

For peer review only

TCs in the two centres have relatively continuous exposure to the intervention over time, allowing them to keep their skills up to date and to ensure the fidelity of the intervention. The fact that the block sizes vary randomly minimizes the possibility of the TCs being able to predict group assignments. Stratification is made by the centre to achieve an equal distribution of patients in the intervention, with control groups at both centres. Furthermore, stratification for sex and HbA1c is crucial to ensure the distribution in the groups due to the knowledge that female adolescents have poorer glycemic control compared with males, and that adolescents with unsatisfactory glycemic control are a high-risk population.[20 21] The grouping for HbA1c is based on ISPAD guidelines 2014.[20] Overall, this approach decreases the within-centre variability and reduces the risk of bias and confounding.[22]An intention-to-treat principle will be used.

Intervention

Transition programme (intervention group)

A multi-component intervention, adapted from the brief transition program of Hilderson et al.[23]and the STEPSTONES-CHD project [16] will be implemented. This transition program comprises eight key components: (1) a transition coordinator (TC); (2) a person-centred written transfer plan; (3) provision of information and education about T1D and treatment, health behaviours, dealing with school, friends, leisure activities and the importance of treatment adherence; (4) high access to the TC via telephone/sms; (5) information about and contact with the adult diabetes care team; (6) meeting with other young people with T1D; (7) support for and guidance of parents; and (8) the actual transfer to the adult diabetes outpatient clinic. The components are implemented in five steps: (1) first visit with TC at the diabetes pediatric outpatient clinic at the age of 16 years; (2) second visit with TC at the diabetes pediatric outpatient clinic at the age of 17 years; (3) "adolescent day" group information with

For peer review only - http://bmjopen.bmj.com/site/about/guidelines.xhtml

parents and adolescents, with the participation of healthcare professionals from the adult outpatient clinic and young adults with T1D; (4) third visit with TC at the diabetes outpatient clinic at the age of 18 years; and (5) the current transfer to one of the eight adult outpatient clinics that care for adults with T1D in the area (Figure 1). The intervention is delivered by specialized trained pediatric nurses at the two pediatric diabetes outpatient clinics (study arm 1 and 2). The content of the patient education is adapted to T1D.

Usual care (intervention and control group)

The current usual care is provided to all participants, irrespective of whether they are randomized to the intervention or not. Usual care includes check-up visits to the paediatric diabetes outpatient clinic according to international, national and local guidelines every third months, with adjustments for individual needs.[24 25]Visits include a meeting at the pediatric diabetes outpatient clinic with a nurse or a physician and it is up to each family if the parents attend these visits. In the area there are several adult diabetes outpatients' clinics to be transferred to. In one of the two intervention clinics usual care includes that adolescents planned to be transferred to the adult diabetes outpatient clinic in the same hospital will be scheduled for a transfer visit in groups. If an adolescent does not participate in this group referral, the same routines apply as for the rest of the adolescents in the control group, i.e., they will be referred directly to the chosen adult diabetes clinic. None of the two intervention centres has a formal TC in place and no additional transfer preparation.

Youth clinic

At the dedicated youth clinic (study arm 3), adolescents with T1D will be followed up from 13 years of age and a more flexible transfer to adult care at 19-20 years of age will be applied.Usual care in this clinic includes visits to the diabetes nurse or the physician at the

For peer review only

unit every second to third month with closer visits if needed, according to the patient's glycemic control. Midwives and social workers are employed at the unit and, if needed, visits to these can be arranged. The midwives provide advice on birth control, STDs and consultations in case of pregnancy. In the case of medical abortion, this can be performed in the youth clinic in a youth-friendly environment. No formal TC is in place, nor a formalized transition/transfer plan.

Intervention fidelity

The special training of TC together with their experience of working with children and adolescents with T1D will ensure compliance that the components are implemented in line with the developed intervention. Prior to the start of this study, the TCs received five days of training including: adolescent psychosocial development and health with focus on chronic diseases in general and T1D in particular; PCC and empowerment; common neuropsychiatric diagnoses; the legal and human rights of young people. Moreover, they received training in communicating and interviewing young people using the psychosocial interview guide HEEADS, the acronym standing for home, education/employment, activities, drugs, sexuality and suicide/depression. The method uses structured questions to facilitate communication and create a sympathetic, confidential, respectful environment.[26]The TC training was provided by experts within each field. TCs were also introduced to the administrative parts of the study in line with Good Clinical Practice (GCP) and have participated in a course in GCP.

Standard Operating Procedures (SOP) were developed to ensure consistency throughout the study. These SOPs include ethical approval, study preparation and study administration, participant recruitment and survey completion; usual care, data storage, entry and security, study progress, process evaluation and publication policy. The Medical Research Council (MRC) guidance on process evaluation of complex interventions is applied to

For peer review only - http://bmjopen.bmj.com/site/about/guidelines.xhtml

monitor intervention fidelity.[27] Acceptability, adherence/fidelity and attrition are monitored using specific assessment forms; enrolment/follow-up form, and intervention implementation form to secure intervention delivery. These are filled out by the TCs during the entire intervention period. Qualitative interviews after completed delivery of the intervention will provide insights into the context of the intervention and the mechanisms of impact, based on the experiences of participants.

To avoid contamination, the TCs will not care for adolescents from the control group or adolescents eligible for the study, and team members at the outpatient clinic who are not involved in the study will only be given a brief outline of the intervention program content.

For peer review only

Data collection

Baseline data will be collected when participants (the adolescent and one or two parents) have agreed to participate in the study. Participants are asked to complete the set of pen-and-paper questionnaires at 16, 17 and 18.5 years of age (Figure 1). These are sent by post to the participants' home address before the next scheduled outpatient visit. The pack contains an instruction letter, a set of questionnaires and a prepaid and addressed reply envelope. Baseline data (T0) will be collected before any intervention is started (study arm 1). For T0, the most recent HbA1c value before inclusion in the study will be used. It is estimated that the questionnaires will take approximately 30 minutes to complete. Participants will be asked to complete the set of questionnairesthemselves and to return them within three weeks. If the questionnaires are not returned within this time frame, patients will be reminded by a new set of questionnaires or asked to complete them while waiting in the waiting room at the next visit.[16 28]

For peer review only - http://bmjopen.bmj.com/site/about/guidelines.xhtml

Outcome measures

The questionnaire obtained at T0 provides background information including: sex, age, education level, born in Sweden or not; adolescent/parents born in Sweden or not; living situation (adolescent)/marital status (guardian); number and order of siblings. For guardians, employment is also required. Person-reported outcome measures are linked to empowerment and PCC by evaluating the personal determinants of generic and illness specific nature (knowledge, self-efficacy and self-management skills).[18]

Primary outcome measures

Empowerment

The primary outcome is empowerment.Empowerment is measured with the Gothenburg Young Persons Empowerment Scale (GYPES).[29] This scale has been tested in a cross-sectional study in order to determine its psychometric properties in adolescents with CHD and T1D and supporting GYPES validity and reliability as a tool for assessing young persons' empowerment.[26] The five subscales measure: (a) self-perceived level of understanding of their disease (knowledge and understanding); (b) the capacity patients have to handle their disease (personal control); (c) the effect their illness has on their lives and sense of self (identity); (d) the capacity to make decisions along with the healthcare professional (shared decision-making); and (e) the ability to share their experiences and help others who are going through a similar situation (enabling others). The primary outcome, analysed at T2, is the total score ranging from 15 to 75 points, with a higher score reflecting a higher level of empowerment.[29]

Secondary outcome measures

The secondary outcome variables chosen are other important developmental tasks in adolescence and emerging adulthood and in relation to T1D. When evaluating interventions

For peer review only - http://bmjopen.bmj.com/site/about/guidelines.xhtml

aimed at improving the outcome of young adults with T1D, some recommended core outcomes are set.[30]These eight outcomes are: measures of diabetes-related burden or stress, diabetes-related quality of life, number of severe hypoglycemic events, self-management behaviour; number of instances of diabetic ketoacidosis (DKA), objectively-measured glycated hemoglobin (HbA1C), level of clinic engagement and perceived level of control over diabetes. In addition, we measure indicators for successful transition and transfer outcomes as proposed by Coyne et al.,[31] which include clinic attendance, hospitalization rates, disease-specific outcomes and transfer-specific satisfaction. See Table 1 for an overview of included variables and measurements. Secondary outcomes include endpoint at T2 and change from baseline to T2 (also empowerment), while the subscales in questionnaires will be included in exploratory analyses.

Generic

Health behaviours are measured with the Health Behaviour Scale. This questionnaire has been validated in adolescents with CHD but is also considered to be valid for the diabetes group. Health behaviours are ‘activities a person undertakes to maintain or improve health and prevent diseases. This scale assesses alcohol consumption, tobacco use, dental care and physical activity. The scale has 15 items that help to calculate three summary risk scores: substance use (score 0–100), dental hygiene (score 0–100) and total health risk score (score 0–100). Higher risk scores represent unhealthier behaviors.[32]

Diabetes specific

Diabetes duration, Insulin administration (Multiple Daily Injections, MDI or Continuous Subcutaneous Insulin Infusion, CSII), Blood glucose testing (Self-Monitoring Blood Glucose testing, SMBG/Continuous Glucose Monitoring CGM/Intermittent scanning Continuous Glucose Monitoring, isCGM) are descriptive variables. Hypoglycemic events and diabetic

For peer review only - http://bmjopen.bmj.com/site/about/guidelines.xhtml

ketoacidosis (DKA) will be summed up for the whole study period and compared at T2. HbA1c will be analysed using DCA Vantage (Siemens Healthcare Diagnostics AB, UpplandsVäsby, Sweden) and the test results collected from the National Diabetes Register (NDR). Analyses will be performed at T2 and change from baseline to T2.

'Check your Health' measures self-perceived physical and emotional health, social relationships and general QoL on four vertical thermometer scales ranging from 0 to 100, with 0 indicating low self-perceived health. Each scale indicates self-perceived health with diabetes and, on the same scale, self-perceived health imagined without diabetes. The measured difference between self-perceived health with and the imagined without diabetes is defined as the burden of diabetes.[33]

DISABKIDS Chronic Generic Measure - DCGM-12 measures general quality of life and the level of distress caused by a chronic disease. The short-form of the DISABKIDS chronic generic module consists of 12 five-point Likert-scaled items assigned to the three domains: mental, social and physical. Higher scores represent better outcome.[34-36]

DISABKIDS Diabetes Module (DM) consists of 13 five-point Likert scaled items and has two scales: an Impact and a Treatment scale. The Impact scale describes emotional reactions of the need to control everyday life and restrictions of one's diet. The Treatment scale refers to carrying equipment and planning treatment. Higher scores represent better outcome.[34 36 37]

Transfer specific

The Readiness for transition questionnaire RTQ (adolescent and parent version) examines two aspects. Firstly, the overall transition readiness is assessed, using two items ranging from 1 to 4. The sum of these two items results in a score ranging from 2 to 8. Secondly, the frequency

For peer review only - http://bmjopen.bmj.com/site/about/guidelines.xhtml

of adolescent responsibility and parental involvement are reported for 10 different health behaviours on a four-point Likert scale. Each variable results in a total score ranging from 10 to 40, with higher scores indicating higher adolescent responsibility or parental involvement respectively.[38]

Parental uncertainty is measured using a 10 cm Linear Analogue Scale (LAS) with the end points 'not uncertain at all' (=0) and 'extremely uncertain' (=100). Parental uncertainty comprehends parents' perceptions of certainty/uncertainty prior to their child's transfer to adult care. This LAS has been used in a cross-sectional study of adolescents with CHD, was developed for an ongoing study in the STEPSTONES-CHD project and has been used in a cross-sectional study.[39]

The new questionnaire on transfer and transition experiences is under construction and will be tested on young persons aged 19-21 years with long-term conditions and experience of the transfer to adult care in order to evaluate its psychometric properties.

Clinical indicators, such as attendance for scheduled outpatient visits before and after transfer to adult clinic and the duration between last visit in pediatric care and the first in adult care will be collected from the medical record.

Information about validity, reliability and responsiveness of the questionnaires is provided in a supplemental file.[29 32-35 37-40]

Table 1 Overview of variables and measurements in Stepstones-DIAB

**Variables/indicators**	**Measurement**	**Time point**	**Source**
**PRIMARY OUTCOME**			
Patient empowerment	Gothenburg Young Persons Empowerment scale (GYPES-DIAB) [29]	T0, T1, T2	A
**SECONDARY OUTCOMES**			

For peer review only - http://bmjopen.bmj.com/site/about/guidelines.xhtml

**Generic**			
Health behavior	Health Behavior Scale[41]	T0, T1, T2	A
**Diabetes-specific**	Diabetes duration<br>Insulin administration (MDI/CSII)<br>Blood glucose testing (SMBG/CGM/isCGM)	T0	NDR
Glycemic control	HbA1c, hypoglycemic events, diabetic ketoacidosis	T0, T1, T2	NDR
Diabetes burden	Check yourhealth[33]	T0, T1, T2	A
HRQoL /level of control	DisabKids Chronic Generic Measure-12 [34-36]<br>Disabkids Diabetes Module-10 [34 36 37]	T0, T1, T2	A
**Transfer-specific**			
Transition readiness	Readiness for Transition Questionnaire (RTQ) [38]	T0, T1, T2	A + P
Uncertainty (parents)	Uncertainty Scale (Linear Analog Scale) [39]	T0, T1, T2	P
Clinical indicators	Clinic attendance rates, pre- and post-transfer<br>Time between last visit in PC and first visit in AC	T2	Admin
Transitional Care experiences Questionnaire	In progress	T2	A

A= Adolescents, P= Parents, NDR= National Diabetes Registry, PC=Pediatric care, AC= Adult care

Data management

The completed questionnaires are entered into a secure research database, using a web-based data entry system, accessible for research group only. Each patient is assigned a unique study codeand this code will be used as a unique identifier in the database to ensure patient anonymity. The list of study codes and the list of eligible participants will be stored separately in a secure file to which only the TC coordinators have access. In study arm 3, a data collection officer will handle this process.

Data monitoring and quality checks will be undertaken by a team member with vast experience of database management. To make data collection procedures uniform, TCs and the DCO have been provided with thorough training; regular visits will be made by the

For peer review only

project coordinator and regular quality assurance sessions will be organized for TCs and DCO.

Owing to the nature of the intervention and the study design, it is not possible to blind the participants but TCs in charge of carrying out the intervention and data collection are not involved in the preparation of the intervention design or in the statistical analyses.

Proposed sample size

Based on the primary outcome, we target an improved patient empowerment score of 5.25 points on a scale from 15-75 (i.e. 0.5 SD).[29] For two-sided tests with alpha=0.05 and power=80%, 63 patients are needed in each arm of the RCT. In order to compensate for a potential 10% dropout rate, 70 patients will be included in each arm. Given that the two pediatric diabetes outpatient clinics together have about 100 patients in each age cohort of adolescents, and considering a 70% participation rate, a 24-month recruitment period is needed to enrol 140 patients in study arm 1 and 2.

Data analysis

The primary endpoint (total score of the level of empowerment at T2) will be analysed using Fisher's non-parametric permutation test unadjusted between the intervention group and the control group in the RCT on the Full Analysis Set with imputation of missing values using stochastic imputation. A sensitivity analysis will be conducted with ANCOVA adjusting for stratification variables without imputation. If confounders are found, complementary analyses will be conducted using ANCOVA between the two groups adjusted for these confounders. A confounder is a baseline variable that differs between the groups and influences the primary outcome variable. For comparison between the two randomized groups, Fisher's non-

For peer review only - http://bmjopen.bmj.com/site/about/guidelines.xhtml

parametric permutation test will be used for continuous variables, Fisher's exact test for dichotomous variables, Mantel-Haenszel chi-square test for ordered categorical variables and Pearson Chi-square test for non-ordered categorical variables. Missing data will be handled with stochastic imputation. Mean difference between the two groups with 95% CI will be presented for all continuous and dichotomous variables. For comparison within groups, Fisher's non-parametric permutation test for paired observation will be used for continuous variables and Sign test for dichotomous variables and ordered categorical variables.

All analyses will be predefined in a detailed statistical analysis plan (SAP) before data base lock. In a later stage, a comparison between the intervention group and a youth clinic group (study arm 3) will also be performed. All significance tests will be two-sided and conducted at the 5% significance level.

Patient and public involvement

During the planning phase of this study, young persons from the Swedish patient organization, "Young Diabetes", have been involved as advisors in the development of certain parts of the intervention. Insufficient transitional care has been on their agenda for several years and the initiative to evaluate support for transition and transfer is therefore highly sought after. Representatives from Young Diabetes will continuously be involved as a reference group during the entire study to secure consideration of young peoples' priorities, experiences and preferences in all phases. These representatives also participate in the event "Adolescent Day". There is an established advisory board in STEPSTONES that has been involved in the development of the intervention, also including young persons with CHD and parent representatives.[16] Henceforth, this advisory board will serve the entire STEPSTONES project, and thus young persons with diabetes have been included in the advisory board.

For peer review only - http://bmjopen.bmj.com/site/about/guidelines.xhtml

Further, this group includes experts on adolescent health and medicine, as well as on the research and implementation of PCC.

Trial duration

Recruitment of participants began in August 2019 and is expected to be completed in August 2021. Data collection is planned to be completed in January 2024. When data collection is completed, the dataset will be closed and the analysis of data and the dissemination of the results will begin.

Ethical considerations

For this study, Ethics approval has been obtained from the Regional Ethics Review Board in Stockholm (Dnr 2018/1725-31). A multi-centre ethical application includes mandatory approvals from the head of the department at each participating centre.

Before inclusion, an information pack is given to the adolescents and their parents to explain the purpose of the study, its voluntary nature, the right to cease participation at any time, the data storage procedure, and the strict anonymous processing of data. Patients will only be included if the adolescent, who is a minor at the time of inclusion, and the parents/guardians provide written informed consent.

The risk of participating in this study is low, as the intervention is supportive in nature. If the transition programme is shown to be effective, the benefits may be substantial. We consider the participants to be vulnerable persons. For some participants, filling out the questionnaires may bring up some unpleasant memories and/or feelings, so contact details of the TCs transition coordinators/data collection officers, as well as organisations which can help them

For peer review only

cope with these feelings, will be provided in the information pack. Participants are covered by Swedish patient insurance in case of adverse events.

Follow-up on adverse events

All adverse events (AE) will be reported within a week to the principal investigator and the project manager. In case of a serious AE, the report will be made immediately. Appropriate measures will be undertaken in every AE to ensure patent safety.

Dissemination

The results from this study will be published in peer-reviewed journals and will follow the recommendations of the CONSORT statement.[40] Further, abstracts for poster and oral presentation will be submitted to national and international conferences and findings communicated and discussed with health care and patient representatives for potential implementation.

DISCUSSION

This study relies on the experiences from two previous research projects aiming to promote the transition process for youths. The first instance was for adolescents with rheumatoid arthritis, the second is the current STEPSTONES-CHD research project by testing a further developed transition program in adolescents with CHD,[16 42] and now we are expanding to evaluate the effectiveness of a structured, person-centred transition programme for adolescents with T1D. The focus is to increase their level of empowerment in order to

For peer review only - http://bmjopen.bmj.com/site/about/guidelines.xhtml

promote their ability to navigate through the adult care system, participate in care planning, and increase self-management and decision-making skills.[6 7]

As previously stated, creating effective clinical processes for the transition from pediatric to adult care is particularly important in order to optimize well-being and health in emerging adults with T1D. This includes achieving target glycemic control to prevent long-term complications and to maximize lifelong functioning. Although there is a lack of proven strategies to achieve these goals, programs that particularly target adolescents with T1D through education, skills training, special transition clinics, and/or addition of TCs appear to be promising.[10 43]However, the current knowledge regarding this transition process calls for ongoing and expanding research initiatives in order to determine the effectiveness of transition programs for adolescents with different long term conditions.[9 44]

One of the strengths of this study is the chosen design; employing an RCT is considered the gold standard when evaluating the effectiveness of interventions and provides the highest level of evidence. By using stratified block randomisation, we ensure that the intervention and control groups are balanced in terms of potential confounding factors, e.g. equal distribution, thereby minimising the risk of biasing the result. In a previous empowerment-based study including a person-centred group education for youths with T1D, the results highlighted the importance of an approach that strengthens young people's independence and autonomy. The parents' attitude was also highlighted in this process. The adolescents described the importance of a permissive atmosphere and that it was perceived as positive to meet at new healthcare professional in these encounters. Moreover, the adolescents said it was empowering to meet others with T1D.[45] For HbA1c, an effect could only be seen for boys.[46] Another strength is that projects can use the lessons learned from STEPSTONES-CHD in terms of feasibility and fidelity. For example, experiences of standard operating procedures for recruitment and data collection will secure data entry. Moreover, in the

For peer review only - http://bmjopen.bmj.com/site/about/guidelines.xhtml

training of the TCs, the experienced TCs from STEPSTONES-CHD could be used as tutors and mentors, which further secures delivery of the generic intervention components in STEPSTONES-DIAB. By employing experienced diabetes nurses as TCs, we ensure that the generic components are adapted to and relevant for diabetes care. Further, the generic components in the person-centred transition program, as well as the generic outcome measures, provide opportunities to compare findings across different conditions. If the program is proven effective, these circumstances may facilitate future implementation of transfer and transition strategies in clinical practice.

A challenge in complex interventions like transition programs is the multiple interacting components and behaviours required by those delivering and receiving the intervention. To evaluate the full extent of a complex intervention in addition to effectiveness, the evaluation must incorporate process assessments of how the intervention is being delivered in practice and what causal mechanisms lead to the desired outcomes. Based on the experiences from the more extensive process evaluation undertaken in STEPSTONES-CHD,[17] we have identified certain process evaluation components that also need to be undertaken in STEPSTONES-DIAB in order to promote future implementation. These components reflect what is delivered (fidelity, adherence, dose, reach, acceptability) and are provided through participant observations, the intervention implementation form, enrolment form and transition plans.[17]

Some limitations need to be acknowledged in this study. Firstly, this intervention focuses on preparation for transition to adulthood and the actual transfer to adult care. Additional interventions may be needed to secure continuity of care after transfer. Secondly, the lack of blinding procedures poses a limitation in that the nature of study makes blinding patients and TCs impossible. Thirdly, the short follow-up period of six months at the age of 18.5 years cannot be considered enough to evaluate long-term effects of the transition

For peer review only - http://bmjopen.bmj.com/site/about/guidelines.xhtml

program. There is a great need to evaluate such effects[9]and these must be followed up in separate studies after additional consent and funding.

CONCLUSION

The empirical underpinning of the guidelines, recommendations and statements regarding successful transitional strategies is weak and to be considered expert-based rather than evidence-based. Based on an RCT design and building on previous projects, STEPSTONES-DIAB has the research capacity to provide high-level evidence within this area, and thereby contribute to filling the gaps in scientific knowledge related to transitional care of adolescents with T1D.

Author contributions

CSL, ELB, PM and ALB designed the study. ALB and CSL drafted the manuscript. ELB, PM, AE, EJ, TT participated in reviewing and editing. All authors read and approved the final manuscript.

Funding statement

This work was supported by the Swedish Child Diabetes Foundation (no grant number available), the Swedish Diabetes Association Research Foundation(grant DIA2018-326), Swedish Research Council for Health, Working Life and Welfare-FORTE (grant STYA-2015/0003), Swedish Research Council (grant 2015-02503), the Institute of Health and Care Sciences of the University of Gothenburg and the Gothenburg University Centre for Person-centred care (GPCC).

For peer review only

Competing interests

None declared

For peer review only

For peer review only - http://bmjopen.bmj.com/site/about/guidelines.xhtml

Figure 1 Overview of the study design

REFERENCES

1. International Diabetes Federation (IDF). IDF Diabetes Atlas, 8th edition. 2017
2. Michaud PA, Suris JC, Viner R. The adolescent with a chronic condition. Part II: healthcare provision. *Arch Dis Child* 2004;89(10):943-9. doi: 10.1136/adc.2003.045377 [published Online First: 2004/09/24]
3. Colver A, Longwell S. New understanding of adolescent brain development: relevance to transitional healthcare for young people with long term conditions. *Arch Dis Child* 2013;98(11):902-7. doi: 10.1136/archdischild-2013-303945 [published Online First: 2013/08/30]
4. American Academy of Pediatrics, American Academy of Family Physicians, American College of Physicians-American Society of Internal Medicine. A consensus statement on health care transitions for young adults with special health care needs. *Pediatrics* 2002;110(6 Pt 2):1304-6. [published Online First: 2002/11/29]
5. Suris JC, Belanger RE, Ambresin AE, et al. Extra burden of psychosomatic complaints among adolescents suffering from chronic conditions. *J Dev Behav Pediatr* 2011;32(4):328-31. doi: 10.1097/DBP.0b013e3181fa5727 [published Online First: 2011/04/20]
6. Bravo P, Edwards A, Barr PJ, et al. Conceptualising patient empowerment: a mixed methods study. *BMC Health Serv Res* 2015;15:252. doi: 10.1186/s12913-015-0907-z
7. Ekman I, Swedberg K, Taft C, et al. Person-centered care--ready for prime time. *Eur J Cardiovasc Nurs* 2011;10(4):248-51. doi: S1474-5151(11)00129-0 [pii]10.1016/j.ejcnurse.2011.06.008 [published Online First: 2011/07/19]
8. Small N, Bower P, Chew-Graham CA, et al. Patient empowerment in long-term conditions: development and preliminary testing of a new measure. *BMC Health Serv Res* 2013;13:263. doi: 10.1186/1472-6963-13-263 [published Online First: 2013/07/10]
9. Campbell F, Biggs K, Aldiss SK, et al. Transition of care for adolescents from paediatric services to adult health services. *The Cochrane database of systematic reviews* 2016;4:Cd009794. doi: 10.1002/14651858.CD009794.pub2 [published Online First: 2016/04/30]
10. Schultz AT, Smaldone A. Components of Interventions That Improve Transitions to Adult Care for Adolescents With Type 1 Diabetes. *J Adolesc Health* 2017;60(2):133-46. doi: 10.1016/j.jadohealth.2016.10.002 [published Online First: 2016/12/13]
11. Castensoe-Seidenfaden P, Jensen AK, Smedegaard H, et al. Clinical, behavioural and social indicators for poor glycaemic control around the time of transfer to adult care: a longitudinal study of 126 young people with diabetes. *Diabet Med* 2017;34(5):667-75. doi: 10.1111/dme.13318 [published Online First: 2017/01/19]
12. Shulman R, Chafe R, Guttmann A. Transition to Adult Diabetes Care: A Description of Practice in the Ontario Pediatric Diabetes Network. *Canadian journal of diabetes* 2019;43(4):283-89. doi: 10.1016/j.jcjd.2018.10.007 [published Online First: 2019/02/06]
13. O'Hara MC, Hynes L, O'Donnell M, et al. A systematic review of interventions to improve outcomes for young adults with Type 1 diabetes. *Diabet Med* 2017;34(6):753-69. doi: 10.1111/dme.13276
14. Peters A, Laffel L. Diabetes care for emerging adults: recommendations for transition from pediatric to adult diabetes care systems: a position statement of the American Diabetes Association, with representation by the American College of Osteopathic Family Physicians, the American Academy of Pediatrics, the American Association of Clinical Endocrinologists, the American Osteopathic Association, the Centers for Disease Control and Prevention, Children with Diabetes, The Endocrine Society, the International Society for Pediatric and Adolescent Diabetes, Juvenile Diabetes Research Foundation International, the National

For peer review only

Diabetes Education Program, and the Pediatric Endocrine Society (formerly Lawson Wilkins Pediatric Endocrine Society). *Diabetes Care* 2011;34(11):2477-85. doi: 10.2337/dc11-1723 [published Online First: 2011/10/26]

15. Cameron FJ, Garvey K, Hood KK, et al. ISPAD Clinical Practice Consensus Guidelines 2018: Diabetes in adolescence. *Pediatr Diabetes* 2018;19 Suppl 27:250-61. doi: 10.1111/pedi.12702 [published Online First: 2018/06/15]
16. Acuna Mora M, Sparud-Lundin C, Bratt EL, et al. Person-centred transition programme to empower adolescents with congenital heart disease in the transition to adulthood: a study protocol for a hybrid randomised controlled trial (STEPSTONES project). *BMJ open* 2017;7(4):e014593. doi: 10.1136/bmjopen-2016-014593 [published Online First: 2017/04/20]
17. Saarijarvi M, Wallin L, Moons P, et al. Transition program for adolescents with congenital heart disease in transition to adulthood: protocol for a mixed-method process evaluation study (the STEPSTONES project). *BMJ open* 2019;9(8):e028229. doi: 10.1136/bmjopen-2018-028229 [published Online First: 2019/08/05]
18. Acuna Mora M, Saarijarvi M, Sparud-Lundin C, et al. Empowering Young Persons with Congenital Heart Disease: Using Intervention Mapping to Develop a Transition Program - The STEPSTONES Project. *J Pediatr Nurs* 2020;50:e8-e17. doi: 10.1016/j.pedn.2019.09.021 [published Online First: 2019/11/02]
19. Chan AW, Tetzlaff JM, Altman DG, et al. SPIRIT 2013 Statement: defining standard protocol items for clinical trials. *Rev Panam Salud Publica* 2015;38(6):506-14. [published Online First: 2016/07/22]
20. Rewers MJ, Pillay K, de Beaufort C, et al. Assessment and monitoring of glycemic control in children and adolescents with diabetes. *Pediatr Diabetes* 2014;15 Suppl 20:102-14. doi: 10.1111/pedi.12190
21. The Swedish Pediatric Diabetes Quality Registry, SWEDIABKIDS. Swediabkids - The National Quality Register for Child and Adolescent Diabetes Annual Report 2018. 2019 [Available from: https://www.ndr.nu accessed 11 October 2019.
22. Efird J. Blocked randomization with randomly selected block sizes. *Int J Environ Res Public Health* 2011;8(1):15-20. doi: 10.3390/ijerph8010015 [published Online First: 2011/02/15]
23. Hilderson D, Westhovens R, Wouters C, et al. Rationale, design and baseline data of a mixed methods study examining the clinical impact of a brief transition programme for young people with juvenile idiopathic arthritis: the DON'T RETARD project. *BMJ open* 2013;3(12):e003591. doi: 10.1136/bmjopen-2013-003591 [published Online First: 2013/12/05]
24. Forsberg M, Forsander G, , Reference group. Diabetes at outpatient clinics for children and adolescents; Guidelines from the paediatrician association for endocrinology and diabetes in Sweden 2017 [Available from: http://endodiab.barnlakarforeningen.se/vardprogram/diabetes/ accessed 11 October 2019 2019.
25. Pihoker C, Forsander G, Fantahun B, et al. ISPAD Clinical Practice Consensus Guidelines 2018: The delivery of ambulatory diabetes care to children and adolescents with diabetes. *Pediatr Diabetes* 2018;19 Suppl 27:84-104. doi: 10.1111/pedi.12757 [published Online First: 2018/08/26]
26. Goldenring JM, E C. Getting into adolescent heads. *Contemp Pediatr* 1988
27. Moore GF, Audrey S, Barker M, et al. Process evaluation of complex interventions: Medical Research Council guidance. *BMJ* 2015;350:h1258. doi: 10.1136/bmj.h1258 [published Online First: 2015/03/21]
28. Apers S, Moons P, Goossens E, et al. Sense of coherence and perceived physical health explain the better quality of life in adolescents with congenital heart disease. *Eur J Cardiovasc Nurs* 2013;12(5):475-83. doi: 10.1177/1474515113477955 [published Online First: 2013/02/15]

For peer review only

29. Acuna Mora M, Luyckx K, Sparud-Lundin C, et al. Patient empowerment in young persons with chronic conditions: Psychometric properties of the Gothenburg Young Persons Empowerment Scale (GYPES). *PLoS One* 2018;13(7):e0201007. doi: 10.1371/journal.pone.0201007 [published Online First: 2018/07/22]
30. Byrne M, O'Connell A, Egan AM, et al. A core outcomes set for clinical trials of interventions for young adults with type 1 diabetes: an international, multi-perspective Delphi consensus study. *Trials* 2017;18(1):602. doi: 10.1186/s13063-017-2364-y [published Online First: 2017/12/21]
31. Coyne B, Hallowell SC, Thompson M. Measurable Outcomes After Transfer From Pediatric to Adult Providers in Youth With Chronic Illness. *J Adolesc Health* 2017;60(1):3-16. doi: 10.1016/j.jadohealth.2016.07.006 [published Online First: 2016/09/12]
32. Goossens E, Luyckx K, Mommen N, et al. Health risk behaviors in adolescents and emerging adults with congenital heart disease: psychometric properties of the Health Behavior Scale-Congenital Heart Disease. *Eur J Cardiovasc Nurs* 2013;12(6):544-57. doi: 10.1177/1474515113475934
33. Viklund G, Wikblad K. Self-perceived health and burden of diabetes in teenagers with type 1 diabetes: psychometric properties of the Swedish measure 'check your health'. *Acta Paediatr* 2010;99(3):422-6. doi: APA1615 [pii]10.1111/j.1651-2227.2009.01615.x [published Online First: 2009/12/05]
34. Bullinger M, Schmidt S, Petersen C. Assessing quality of life of children with chronic health conditions and disabilities: a European approach. *Int J Rehabil Res* 2002;25(3):197-206. [published Online First: 2002/09/28]
35. Simeoni MC, Schmidt S, Muehlan H, et al. Field testing of a European quality of life instrument for children and adolescents with chronic conditions: the 37-item DISABKIDS Chronic Generic Module. *Qual Life Res* 2007;16(5):881-93. doi: 10.1007/s11136-007-9188-2 [published Online First: 2007/04/04]
36. Chaplin JE, Hallman M, Nilsson NO, et al. The reliability of the disabled children's quality-of-life questionnaire in Swedish children with diabetes. *Acta Paediatr* 2012;101(5):501-6. doi: 10.1111/j.1651-2227.2011.02581.x [published Online First: 2012/01/04]
37. Baars RM, Atherton CI, Koopman HM, et al. The European DISABKIDS project: development of seven condition-specific modules to measure health related quality of life in children and adolescents. *Health and quality of life outcomes* 2005;3:70. doi: 1477-7525-3-70 [pii]10.1186/1477-7525-3-70 [published Online First: 2005/11/15]
38. Gilleland J, Amaral S, Mee L, et al. Getting ready to leave: transition readiness in adolescent kidney transplant recipients. *J Pediatr Psychol* 2012;37(1):85-96. doi: 10.1093/jpepsy/jsr049 [published Online First: 2011/09/01]
39. Burstrom A, Acuna Mora M, Ojmyr-Joelsson M, et al. Parental uncertainty about transferring their adolescent with congenital heart disease to adult care. *J Adv Nurs* 2019;75(2):380-87. doi: 10.1111/jan.13852 [published Online First: 2018/09/14]
40. Chaplin JE, Hanas R, Lind A, et al. Assessment of childhood diabetes-related quality-of-life in West Sweden. *Acta Paediatr* 2009;98(2):361-6. doi: 10.1111/j.1651-2227.2008.01066.x
41. Goossens E, Luyckx K, Mommen N, et al. Health risk behaviors in adolescents and emerging adults with congenital heart disease: psychometric properties of the Health Behavior Scale-Congenital Heart Disease. *Eur J Cardiovasc Nurs* 2013;12(6):544-57. doi: 10.1177/1474515113475934 [published Online First: 2013/01/31]
42. Hilderson D, Moons P, Van der Elst K, et al. The clinical impact of a brief transition programme for young people with juvenile idiopathic arthritis: results of the DON'T RETARD project. *Rheumatology (Oxford)* 2016;55(1):133-42. doi: 10.1093/rheumatology/kev284 [published Online First: 2015/09/01]
43. Gabriel P, McManus M, Rogers K, et al. Outcome Evidence for Structured Pediatric to Adult Health Care Transition Interventions: A Systematic Review. *J Pediatr* 2017;188:263-69.e15. doi: 10.1016/j.jpeds.2017.05.066 [published Online First: 2017/07/03]

For peer review only

44. Acuna Mora M, Saarijarvi M, Moons P, et al. The Scope of Research on Transfer and Transition in Young Persons With Chronic Conditions. *J Adolesc Health* 2019 doi: 10.1016/j.jadohealth.2019.07.014 [published Online First: 2019/09/22]
45. Brorsson AL, Lindholm Olinder A, Viklund G, et al. Adolescents' perceptions of participation in group education using the Guided Self-Determination-Young method: a qualitative study. *BMJ Open Diabetes Res Care* 2017;5(1):e000432. doi: 10.1136/bmjdrc-2017-000432 [published Online First: 2017/12/12]
46. Brorsson AL, Leksell J, Andersson Franko M, et al. A person-centered education for adolescents with type 1 diabetes-A randomized controlled trial. *Pediatr Diabetes* 2019 doi: 10.1111/pedi.12888 [published Online First: 2019/07/04]

For peer review only

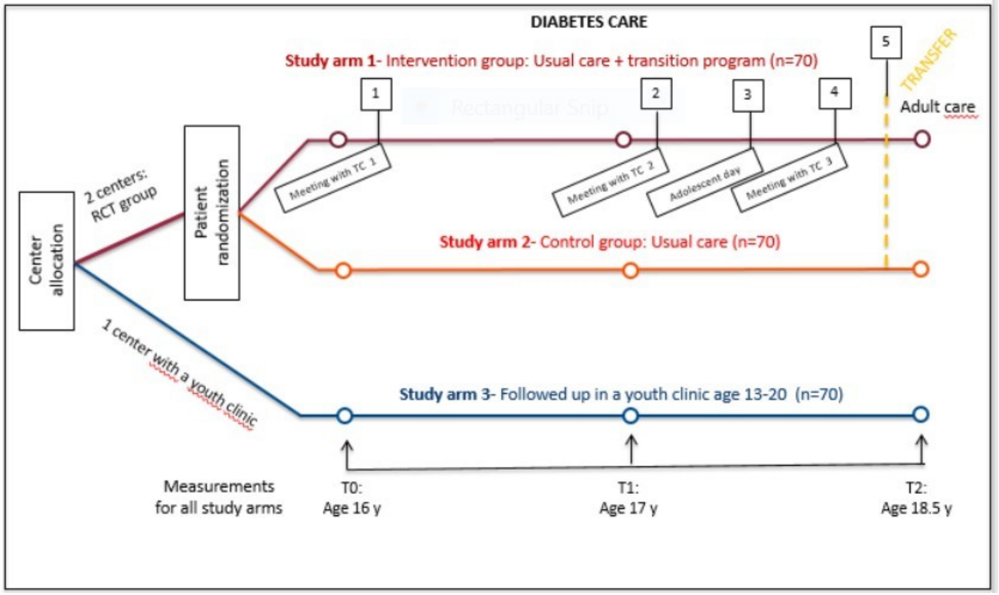

Figure 1 Overview of the study design

Variable	Measurements	Items	Validity	Reliability	Responsiveness	Interpretation
**Primary Outcome**						
Patient empowerment [29]	Gothenburg Young Persons Empowerment Scale (GYPES)	15	Preliminary evidence for content validity and structural validity in young persons with CHD and diabetes. Results from CFA demonstrated that in addition to the subscale scores, an overall empowerment score can be validly computed.	Internal consistency confirmed in young persons with CHD and diabetes with α values of 0.858 for the overall scale and over 0.6 for all subscales.).	As a proxy for assessment of responsiveness, one of the participants had the lowest scoring, only 2.2% had the highest possible score.	Score from 15-75. Higher score reflecting a higher level of patient empowerment.
**Secondary Outcomes**						
Transition readiness [38]	Readiness for Transition Questionnaire (RTQ) adolescent version	26	Validity based on relationships with other variables confirmed in young persons with kidney transplant[38]	Internal consistency confirmed in young persons with kidney transplant with α values over 0.70 [38]	Not reported	Scores from 10-40. Higher scores denote increased adolescent or caregiver responsibility.
Transition readiness [38]	Readiness for Transition Questionnaire (RTQ) parent version	26	Construct validity reported for parents of young persons with kidney transplant.	Reliability reported for parents of young persons with kidney transplant showed α values over 0.70.	Not reported	Scores from 10-40. Higher scores denote increased adolescent or caregiver responsibility.
Health behaviors [41]	Health Behavior Scale-Congenital Heart Disease (HBS-CHD)	15	Item content validity, scale content validity index and validity based on relationships to other variables confirmed in adolescents with CHD. Items deemed relevant for diabetes.	Stability not confirmed.	Confirmed in adolescents with CHD by Guyatt's Responsiveness Index.	Substance use, dental hygiene and total health risk score from 0-100. Physical exercise score from 0-100.
Diabetes burden [33]	Check your health	Perceived physical and emotional health, social relations and general QoL on four vertical scales	Face validity confirmed in adolescents with diabetes, good discriminant validity and moderate convergent validity	Test-retest reliability moderate to good, (i.e. correlation coefficient >0.7) Perceived QoL, emotional health and QoL without diabetes had correlation coefficients just below 0.7.	No floor effects on any of the four health dimensions (0%), ceiling effect (people scoring 100) ranged from 5% to 30%.	Four scales ranging from 0 to 100, higher scores indicating better perceived health ⁄QoL.

For peer review only - http://bmjopen.bmj.com/site/about/guidelines.xhtml

HRQOL [35 36]	DisabKids Chronic Generic Measure-12	12	Construct validity as well as convergent and discriminant validity of the DCGM-37 confirmed. [35]<br><br>No information found on validity for the short version.	Good reliability was found in a Swedish population of children with diabetes, α values of 0.932. Test-retest ICC coefficients were 0.913 and split-half correlation was 0.930. [36]<br><br>No information found on reliability for the short version.	None reached the floor of the scale. In total, 6% of the children answered at the top of a scale. [36]	Total score is 12–60. Higher scores indicating better HRQOL.
Level of control [36 37]	Disabkids Diabetes Module-10	10	Face and content validity confirmed in children with diabetes.[37]	Internal consistency confirmed in young persons with diabetes with α values of 0.83 and 0.84 for the two subscales.[37] Confirmed in a Swedish population, α values of 0.85, test-retest ICC coefficients were 0.913 and split-half correlation 0.848. [36]	None reached the floor of the scale. In total, 6% of the children answered at the top of a scale.[36]	Total score is 10–50. Higher scores indicate higher level of control.
Parental uncertainty [39]	Linear analog scale (LAS)	1<br>+ alternative option: "I have not thought about the transfer"	Face validity confirmed in parents to adolescents with CHD.	Under investigation		Score is from 0 (very uncertain about the transfer to adult care) to 100 (not uncertain about the transfer of care).
Satisfaction with care	Transitional Care Experiences Questionnaire	17<br>+ 3 open ended question	Under investigation			Score from 17-85. Higher score reflecting a higher level of satisfaction with transitional care

For peer review only

Reporting checklist for protocol of a clinical trial.

Based on the SPIRIT guidelines.

Instructions to authors

Complete this checklist by entering the page numbers from your manuscript where readers will find each of the items listed below.

Your article may not currently address all the items on the checklist. Please modify your text to include the missing information. If you are certain that an item does not apply, please write "n/a" and provide a short explanation.

Upload your completed checklist as an extra file when you submit to a journal.

In your methods section, say that you used the SPIRITreporting guidelines, and cite them as:

Chan A-W, Tetzlaff JM, Altman DG, Laupacis A, Gøtzsche PC, Krleža-Jerić K, Hróbjartsson A, Mann H, Dickersin K, Berlin J, Doré C, Parulekar W, Summerskill W, Groves T, Schulz K, Sox H, Rockhold FW, Rennie D, Moher D. SPIRIT 2013 Statement: Defining standard protocol items for clinical trials. Ann Intern Med. 2013;158(3):200-207

		Reporting Item	Page Number
**Administrative information**			
Title	#1	Descriptive title identifying the study design, population, interventions, and, if applicable, trial acronym	1
Trial registration	#2a	Trial identifier and registry name. If not yet registered, name of intended registry	3
Trial registration: data set	#2b	All items from the World Health Organization Trial Registration Data Set	7-21
Protocol version	#3	Date and version identifier	NA, First version
Funding	#4	Sources and types of financial, material, and other support	24

For peer review only

Roles and responsibilities: contributorship	#5a	Names, affiliations, and roles of protocol contributors	1-2, 24
Roles and responsibilities: sponsor contact information	#5b	Name and contact information for the trial sponsor	24, only funding agencies involved, no sponsors
Roles and responsibilities: sponsor and funder	#5c	Role of study sponsor and funders, if any, in study design; collection, management, analysis, and interpretation of data; writing of the report; and the decision to submit the report for publication, including whether they will have ultimate authority over any of these activities	24, funders have no role in these matters.
Roles and responsibilities: committees	#5d	Composition, roles, and responsibilities of the coordinating centre, steering committee, endpoint adjudication committee, data management team, and other individuals or groups overseeing the trial, if applicable (see Item 21a for data monitoring committee)	17, 19
**Introduction**			
Background and rationale	#6a	Description of research question and justification for undertaking the trial, including summary of relevant studies (published and unpublished) examining benefits and harms for each intervention	5-7
Background and rationale: choice of comparators	#6b	Explanation for choice of comparators	5-7
Objectives	#7	Specific objectives or hypotheses	7
Trial design	#8	Description of trial design including type of trial (eg, parallel group, crossover, factorial, single group), allocation ratio, and framework (eg, superiority, equivalence, non-inferiority, exploratory)	7-8

For peer review only

**Methods: Participants, interventions, and outcomes**			
Study setting	#9	Description of study settings (eg, community clinic, academic hospital) and list of countries where data will be collected. Reference to where list of study sites can be obtained	7
Eligibility criteria	#10	Inclusion and exclusion criteria for participants. If applicable, eligibility criteria for study centres and individuals who will perform the interventions (eg, surgeons, psychotherapists)	8, 10-12
Interventions: description	#11a	Interventions for each group with sufficient detail to allow replication, including how and when they will be administered	10-11
Interventions: modifications	#11b	Criteria for discontinuing or modifying allocated interventions for a given trial participant (eg, drug dose change in response to harms, participant request, or improving / worsening disease)	NA
Interventions: adherance	#11c	Strategies to improve adherence to intervention protocols, and any procedures for monitoring adherence (eg, drug tablet return; laboratory tests)	11
Interventions: concomitant care	#11d	Relevant concomitant care and interventions that are permitted or prohibited during the trial	7
Outcomes	#12	Primary, secondary, and other outcomes, including the specific measurement variable (eg, systolic blood pressure), analysis metric (eg, change from baseline, final value, time to event), method of aggregation (eg, median, proportion), and time point for each outcome. Explanation of the clinical relevance of chosen efficacy and harm outcomes is strongly recommended	12-17
Participant timeline	#13	Time schedule of enrolment, interventions (including any run-ins and washouts), assessments, and visits for participants. A	8-9 (Figure 1)

For peer review only

		schematic diagram is highly recommended (see Figure)	
Sample size	#14	Estimated number of participants needed to achieve study objectives and how it was determined, including clinical and statistical assumptions supporting any sample size calculations	18
Recruitment	#15	Strategies for achieving adequate participant enrolment to reach target sample size	8, 18
**Methods: Assignment of interventions (for controlled trials)**			
Allocation: sequence generation	#16a	Method of generating the allocation sequence (eg, computer-generated random numbers), and list of any factors for stratification. To reduce predictability of a random sequence, details of any planned restriction (eg, blocking) should be provided in a separate document that is unavailable to those who enrol participants or assign interventions	8-9
Allocation concealment mechanism	#16b	Mechanism of implementing the allocation sequence (eg, central telephone; sequentially numbered, opaque, sealed envelopes), describing any steps to conceal the sequence until interventions are assigned	8-9
Allocation: implementation	#16c	Who will generate the allocation sequence, who will enrol participants, and who will assign participants to interventions	8
Blinding (masking)	#17a	Who will be blinded after assignment to interventions (eg, trial participants, care providers, outcome assessors, data analysts), and how	18 (NA)
Blinding (masking): emergency unblinding	#17b	If blinded, circumstances under which unblinding is permissible, and procedure for revealing a participant's allocated intervention during the trial	NA

For peer review only

Methods: Data collection, management, and analysis

Data collection plan	#18a	Plans for assessment and collection of outcome, baseline, and other trial data, including any related processes to promote data quality (eg, duplicate measurements, training of assessors) and a description of study instruments (eg, questionnaires, laboratory tests) along with their reliability and validity, if known. Reference to where data collection forms can be found, if not in the protocol	12-17
Data collection plan: retention	#18b	Plans to promote participant retention and complete follow-up, including list of any outcome data to be collected for participants who discontinue or deviate from intervention protocols	12
Data management	#19	Plans for data entry, coding, security, and storage, including any related processes to promote data quality (eg, double data entry; range checks for data values). Reference to where details of data management procedures can be found, if not in the protocol	17
Statistics: outcomes	#20a	Statistical methods for analysing primary and secondary outcomes. Reference to where other details of the statistical analysis plan can be found, if not in the protocol	18-19
Statistics: additional analyses	#20b	Methods for any additional analyses (eg, subgroup and adjusted analyses)	18-19
Statistics: analysis population and missing data	#20c	Definition of analysis population relating to protocol non-adherence (eg, as randomised analysis), and any statistical methods to handle missing data (eg, multiple imputation)	18-19

Methods: Monitoring

For peer review only

Data monitoring: formal committee	#21a	Composition of data monitoring committee (DMC); summary of its role and reporting structure; statement of whether it is independent from the sponsor and competing interests; and reference to where further details about its charter can be found, if not in the protocol. Alternatively, an explanation of why a DMC is not needed	NA
Data monitoring: interim analysis	#21b	Description of any interim analyses and stopping guidelines, including who will have access to these interim results and make the final decision to terminate the trial	NA
Harms	#22	Plans for collecting, assessing, reporting, and managing solicited and spontaneously reported adverse events and other unintended effects of trial interventions or trial conduct	20
Auditing	#23	Frequency and procedures for auditing trial conduct, if any, and whether the process will be independent from investigators and the sponsor	17, funders have no role in these matters.
**Ethics and dissemination**			
Research ethics approval	#24	Plans for seeking research ethics committee / institutional review board (REC / IRB) approval	20
Protocol amendments	#25	Plans for communicating important protocol modifications (eg, changes to eligibility criteria, outcomes, analyses) to relevant parties (eg, investigators, REC / IRBs, trial participants, trial registries, journals, regulators)	Protocol modifications will be communicated in the trial registry
Consent or assent	#26a	Who will obtain informed consent or assent from potential trial participants or authorised surrogates, and how (see Item 32)	8, 20
Consent or assent: ancillary studies	#26b	Additional consent provisions for collection and use of participant data and biological specimens in ancillary studies, if applicable	23

Confidentiality	#27	How personal information about potential and enrolled participants will be collected, shared, and maintained in order to protect confidentiality before, during, and after the trial	17
Declaration of interests	#28	Financial and other competing interests for principal investigators for the overall trial and each study site	24
Data access	#29	Statement of who will have access to the final trial dataset, and disclosure of contractual agreements that limit such access for investigators	Only members of the research group have access to the final dataset, as specified in the approved ethical application.
Ancillary and post trial care	#30	Provisions, if any, for ancillary and post-trial care, and for compensation to those who suffer harm from trial participation	NA
Dissemination policy: trial results	#31a	Plans for investigators and sponsor to communicate trial results to participants, healthcare professionals, the public, and other relevant groups (eg, via publication, reporting in results databases, or other data sharing arrangements), including any publication restrictions	21
Dissemination policy: authorship	#31b	Authorship eligibility guidelines and any intended use of professional writers	No
Dissemination policy: reproducible research	#31c	Plans, if any, for granting public access to the full protocol, participant-level dataset, and statistical code	No
**Appendices**			
Informed consent materials	#32	Model consent form and other related documentation given to participants and authorised surrogates	Only available in Swedish
Biological specimens	#33	Plans for collection, laboratory evaluation, and storage of biological specimens for genetic or	NA

For peer review only

molecular analysis in the current trial and for future use in ancillary studies, if applicable

None The SPIRIT checklist is distributed under the terms of the Creative Commons Attribution License CC-BY-ND 3.0. This checklist can be completed online using https://www.goodreports.org/, a tool made by the EQUATOR Network in collaboration with Penelope.ai

For peer review only
